# Measuring compartmental $T_{2}$-orientational dependence in human brain white matter using a tiltable RF coil and diffusion-$T_{2}$ correlation MRI

**DOI:** 10.1016/j.neuroimage.2021.117967

**Published:** 2021-08-01

**Authors:** Chantal M.W. Tax, Elena Kleban, Maxime Chamberland, Muhamed Baraković, Umesh Rudrapatna, Derek K. Jones

**Affiliations:** a Cardiff University Brain Research Imaging Centre (CUBRIC), School of Physics and Astronomy, Cardiff University, Cardiff, UK; b University Medical Center Utrecht, Utrecht University, Utrecht, the Netherlands; c Cardiff University Brain Research Imaging Centre (CUBRIC), School of Psychology, Cardiff University, Cardiff, UK; d Signal Processing Laboratory 5, Ecole Polytechnique Federale de Lausanne, Lausanne, Switzerland; e Translational Imaging in Neurology Basel, Department of Biomedical Engineering, University Hospital Basel, Basel, Switzerland; f Mary MacKillop Institute for Health Research, Faculty of Health Sciences, Australian Catholic University, Melbourne, Australia

**Keywords:** Diffusion MRI, Microstructure, $T_{2}$ relaxation, Directional anisotropy, Myelin susceptibility

## Abstract

The anisotropy of brain white matter microstructure manifests itself in orientational-dependence of various MRI contrasts, and can result in significant quantification biases if ignored. Understanding the origins of this orientation-dependence could enhance the interpretation of MRI signal changes in development, ageing and disease and ultimately improve clinical diagnosis. Using a novel experimental setup, this work studies the contributions of the intra- and extra-axonal water to the orientation-dependence of one of the most clinically-studied parameters, apparent transverse relaxation $T_{2}$. Specifically, a tiltable receive coil is interfaced with an ultra-strong gradient MRI scanner to acquire multidimensional MRI data with an unprecedented range of acquisition parameters. Using this setup, compartmental $T_{2}$ can be disentangled based on differences in diffusional-anisotropy, and its orientation-dependence further elucidated by re-orienting the head with respect to the main magnetic field ${\vec{B}}_{0}$. A dependence of (compartmental) $T_{2}$ on the fibre orientation w.r.t. ${\vec{B}}_{0}$ was observed, and further quantified using characteristic representations for susceptibility- and magic angle effects. Across white matter, anisotropy effects were dominated by the extra-axonal water signal, while the intra-axonal water signal decay varied less with fibre-orientation. Moreover, the results suggest that the stronger extra-axonal $T_{2}$ orientation-dependence is dominated by magnetic susceptibility effects (presumably from the myelin sheath) while the weaker intra-axonal $T_{2}$ orientation-dependence may be driven by a combination of microstructural effects. Even though the current design of the tiltable coil only offers a modest range of angles, the results demonstrate an overall effect of tilt and serve as a proof-of-concept motivating further hardware development to facilitate experiments that explore orientational anisotropy. These observations have the potential to lead to white matter microstructural models with increased compartmental sensitivity to disease, and can have direct consequences for longitudinal and group-wise $T_{2}$- and diffusion-MRI data analysis, where the effect of head-orientation in the scanner is commonly ignored.

## 1 Introduction

The highly oriented anisotropic architecture of white matter (WM) causes orientation-dependence of various MRI contrasts, such as diffusion-, $T_{2}/T_{2}^{*}/T_{1}$ relaxation-, and magnetisation transfer-weighted MRI (Bender, Klose, 2010, Cherubini, Péran, Hagberg, Varsi, Luccichenti, Caltagirone, Sabatini, Spalletta, 2009, Denk, Torres, MacKay, Rauscher, 2011, Gil, Khabipova, Zwiers, Hilbert, Kober, Marques, 2016, Knight, Damion, Kauppinen, 2018, Knight, Dillon, Jarutyte, Kauppinen, 2017, Knight, Wood, Couthard, Kauppinen, 2015, Lee, van Gelderen, Kuo, Merkle, Silva, Duyn, 2011, Lee, Shmueli, Fukunaga, van Gelderen, Merkle, Silva, Duyn, 2010, Oh, Kim, Cho, Lee, 2013, Pampel, Müller, Anwander, Marschner, Möller, 2015, Rudko, Klassen, De Chickera, Gati, Dekaban, Menon, 2014, Sati, van Gelderen, Silva, Reich, Merkle, De Zwart, Duyn, 2013, Sati, Silva, van Gelderen, Gaitan, Wohler, Jacobson, Duyn, Reich, 2012, Schyboll, Jaekel, Petruccione, Neeb, 2019, Schyboll, Jaekel, Weber, Neeb, 2018, Wharton, Bowtell, 2012, Wharton, Bowtell, 2013, Wiggins, Gudmundsdottir, Le Bihan, Lebon, Chaumeil, 2008). Understanding the origin of this orientational-dependence is of considerable clinical interest because it could enhance the ability to interpret MRI signal changes and, as such, offer improved understanding and ultimately diagnosis of disease. This work focuses on the manifestation of compartmental $T_{2}$- and diffusional-anisotropy in brain WM in diffusion-$T_{2}$ correlation experiments, which simultaneously vary the relaxation- and diffusion-weighting within a single sequence (Barakovic et al., 2021, de Almeida Martins et al., 2020, de Almeida Martins, Tax, Szczepankiewicz, Jones, Westin, Topgaard, 2020, De Santis, Assaf, Jeurissen, Jones, Roebroeck, 2016, Gong et al., 2020, Hutter, Slator, Christiaens, Teixeira, Roberts, Jackson, Price, Malik, Hajnal, 2018, Kleban et al., 2020, Lampinen, Szczepankiewicz, Novén, van Westen, Hansson, Englund, Mårtensson, Westin, Nilsson, 2019, Lampinen, Szczepankiewicz, Mårtensson, van Westen, Hansson, Westin, Nilsson, 2020, Tax, Rudrapatna, Witzel, Jones, 2017, Veraart, Novikov, Fieremans, 2018, Reymbaut et al., 2020).

The diffusion of water molecules is modulated by cellular structures on the micrometer scale and is highly anisotropic in aligned WM fibres because of the directional organisation of the axonal membrane and myelin sheath (Beaulieu, 2002). While myelin contributes to diffusional-anisotropy (Beaulieu, 2002, Jelescu, Zurek, Winters, Veraart, Rajaratnam, Kim, Babb, Shepherd, Novikov, Kim, Fieremans, 2016), the signal from myelin water ($T_{2}∼5-20\;\text{ms}$) has typically decayed away in diffusion MRI experiments due to the comparatively long echo times (TE) employed. The diffusion process can be probed in MRI experiments by varying the orientation, timing, and magnitude of diffusion-encoding gradients (Stejskal and Tanner, 1965) typically in a spin-echo (SE) experiment (Hahn, 1950), and the diffusion-weighted MRI signal varies strongly as a function of fibre-angle with respect to the encoding gradient-orientation (Moseley et al., 1990).

Transverse relaxation involves the loss of phase coherence in the precessional motion of an ensemble of spins (Abragam, 1961, Kowalewski, Mäler, 2018), which can occur due to several mechanisms. In the semiclassical Redfield relaxation theory, the Hamiltonian (the total energy operator) is given as a sum of a large static or time-independent part (typically including the Zeeman term, isotropic chemical shifts, and J-couplings) and a smaller stochastic or time-dependent perturbation (dipolar couplings, quadrupolar couplings for spin angular momentum >1/2, and chemical-shift anisotropy). It is the time-dependent stochastic part of the Hamiltonian that leads to a different time evolution of each spin, ultimately inducing relaxation (with the transverse relaxation commonly summarised by the time constant $T_{2}=1/R_{2}$). However, in a typical free induction decay (FID) experiment conducted with liquids in porous media such as biological tissues, it is not solely the intrinsic $T_{2}$ that determines the rate of signal decay; other mechanisms result in additional dephasing reflected by a shortened *coherence lifetime* (Knight and Kauppinen, 2016). These mechanisms include static effects such as magnetic field inhomogeneities with a characteristic length scale smaller than the macroscopic voxel scale and relaxation ‘sinks’ at the pore surface, and dynamic processes such as exchange between different relaxation domains and diffusion of molecules in the presence of field inhomogeneities. The cumulative observed dephasing in the FID is often summarised in a $T_{2}^{*}$-estimate.

It is well established that in myelinated WM the gradient-recalled-echo (GRE) signal evolution and its magnitude-derived $T_{2}^{*}$ depend on fibre orientation with respect to the main magnetic field ${\vec{B}}_{0}$ (Bender, Klose, 2010, Cherubini, Péran, Hagberg, Varsi, Luccichenti, Caltagirone, Sabatini, Spalletta, 2009, Denk, Torres, MacKay, Rauscher, 2011, Gil, Khabipova, Zwiers, Hilbert, Kober, Marques, 2016, Lee, van Gelderen, Kuo, Merkle, Silva, Duyn, 2011, Lee, Shmueli, Fukunaga, van Gelderen, Merkle, Silva, Duyn, 2010, Oh, Kim, Cho, Lee, 2013, Rudko, Klassen, De Chickera, Gati, Dekaban, Menon, 2014, Sati, van Gelderen, Silva, Reich, Merkle, De Zwart, Duyn, 2013, Sati, Silva, van Gelderen, Gaitan, Wohler, Jacobson, Duyn, Reich, 2012, Wharton, Bowtell, 2012, Wharton, Bowtell, 2013, Wiggins, Gudmundsdottir, Le Bihan, Lebon, Chaumeil, 2008). This orientation-dependence is thought to arise primarily from *static* mesoscopic field inhomogeneities due to magnetic susceptibility effects from the myelin sheath, potentially combined with influences from the vasculature and magic angle dipole-dipole interactions (see Supplementary section 1 for an overview). In contrast, the SE experiment refocuses static field inhomogeneities, and orientation-dependence in the apparent $T_{2}$ may therefore reflect the *dynamic* interplay between diffusion and mesoscopic field inhomogeneities (Carr, Purcell, 1954, Hahn, 1950), as well as magic angle effects in the intrinsic $T_{2}$. As such, the SE experiment offers the opportunity to specifically study features of myelin, exploiting its orientation-dependence, while being less confounded by large-scale static inhomogeneities e.g. from larger vascular structures, air cavities, or suboptimal shimming. In addition, SE experiments have been reported to be more reproducible than GRE (Gil et al., 2016).

Despite initial reports of no apparent $T_{2}$ orientation-dependence in fresh excised bovine WM (Henkelman et al., 1994) at 1.5 T and only subtle effects in fixed human brain WM (Oh et al., 2013) at 7 T, multiple studies have since reported $T_{2}$ anisotropy in the living human brain at 3 T (Gil, Khabipova, Zwiers, Hilbert, Kober, Marques, 2016, Knight, Dillon, Jarutyte, Kauppinen, 2017, Knight, Wood, Couthard, Kauppinen, 2015, McKinnon, Jensen, 2019). Knight et al. (2015) performed $T_{2}$- and diffusion-weighted MRI in two separate experiments (CPMG with shortest TE=24ms and pulsed-gradient SE (PGSE) with b-value $1000\;\text{s}/{\text{mm}}^{2}$), to obtain estimates of $T_{2}$ and the diffusion tensor (DT), respectively. By averaging all $T_{2}$-observations within bins of similarly-oriented fibre-orientation (estimated from the first eigenvector of the DT), $T_{2}$ was found to increase as fibres became more aligned with ${\vec{B}}_{0},$ with this orientation effect being stronger in tissue with higher fractional anisotropy (FA). Gil et al. (2016) used a similar experimental setup (CPMG, shortest TE=9.6ms) and further quantified the isotropic and anisotropic part of $R_{2},$ finding a $\sin^{4}\theta$ dependence on the fibre orientation with respect to ${\vec{B}}_{0},$ θ, in the majority of the regions investigated. Knight et al. (2017) showed that $T_{2}$-anisotropy is differentially affected by age, and linked the observed $\sin^{4}\theta$ angular dependence to the theoretical field-inhomogeneities in the extra-axonal space originating from myelin-susceptibility differences in a hollow-cylinder model (Wharton and Bowtell, 2012).

Even though the hollow-cylinder model compartmentalises the signal into contributions from intra-axonal, extra-axonal, and myelin-water compartments, the vast majority of studies only reported orientation-dependence of the apparent ‘mono-exponential’ $T_{2}^{\left(*\right)},$ i.e., effectively ignoring the fact that the observed signal is an aggregate of those coming from distinct compartments. However, the apparent $T_{2}$ and its orientation-dependence is likely different between compartments (Beaulieu et al., 1998, Birkl et al., 2020, Peled, Cory, Raymond, Kirschner, Jolesz, 1999, Sati, van Gelderen, Silva, Reich, Merkle, De Zwart, Duyn, 2013, Stanisz, Henkelman, 1998), and further disentangling compartmental contributions should enable the formulation of more complete models of WM microstructure.

McKinnon and Jensen (2019) varied the TE in a PGSE sequence while applying a relatively large diffusion-weighting of $b=6000\;\text{s}/{\text{mm}}^{2}$ (shortest TE=90ms) with the aim of nulling the signal from the extra-axonal space and studied $T_{2}$ in the intra-axonal space. Using this setup, together with additional $b=\left[1000,\;2000\right]\;\text{s}/{\text{mm}}^{2}$ measurements at the same TE=90ms, they observed a negative correlation between the intra-axonal $T_{2}$ estimate and the fibre orientation w.r.t. ${\vec{B}}_{0},$ in voxels with a DT-coefficient of linearity (Westin et al., 2002) greater than 0.4. This observation is in apparent contrast with hollow-cylinder models of $T_{2}$-anisotropy, which predict no orientation-dependence of the intra-axonal water $T_{2}$ relaxation time: even though they predict an orientation-dependent frequency offset, the frequency distribution in the intra-axonal space is homogeneous.

This work aims to explore $T_{2}$ anisotropy in different WM compartments using a novel experimental setup. Specifically, a tiltable RF coil, originally designed for patient comfort, was re-purposed and interfaced with a Siemens Connectom scanner, which provides the strongest magnetic field gradients for human MRI experiments currently available on the market at 300mT/m (Jones et al., 2018, Setsompop, Kimmlingen, Eberlein, Witzel, Cohen-Adad, McNab, Keil, Tisdall, Hoecht, Dietz, Cauley, Tountcheva, Matschl, Lenz, Heberlein, Potthast, Thein, Van Horn, Toga, Schmitt, Lehne, Rosen, Wedeen, Wald, 2013) to achieve higher diffusion-weightings at shorter TE than on more commonly-available systems. The bespoke receiver coil can be tilted around the left-right axis by $0^{\circ },$ $9^{\circ }$ and $18^{\circ }$ to ${\vec{B}}_{0},$ which: 1) minimises participant-discomfort when maintaining the head at a fixed angle and thus improves reliability; 2) offers a new degree of freedom, as tilting around the left-right axis is otherwise difficult to achieve; 3) minimises differences in the coil-to-brain distance across different head orientations and thus minimises SNR variations; and therefore 4) increases the repeatability of the experiment. This unique experimental setup allows the acquisition of multidimensional MRI data with an unprecedented range of acquisition parameters (in terms of accessible b-values, TEs, and head-orientations), and can ultimately enable enhanced tissue specificity. To elucidate the origins of $T_{2}$ orientation-dependence in this study, signal contributions from myelin water are minimised by employing sufficiently large TE, and contributions from intra- and extra-axonal water are subsequently disentangled based on differences in their diffusional anisotropy. In addition, confounding effects from misregistrations between modalities and head-orientations are avoided by simultaneously varying diffusion- and $T_{2}$-weighting in diffusion–relaxation correlation acquisitions, and employing a tract-based approach to achieve spatial correspondence. Indeed, the observation of compartmental $T_{2}$ orientation-dependence can not only impact our understanding of WM microstructure, but will also have important practical ramifications for the analysis and comparison of $T_{2}$- and dMRI data, where the effect of head-orientation in the scanner, or the relative orientation of structures with respect to each other even in a static head, is commonly ignored.

## 2 Methods and materials

### 2.1 Data acquisition

**Fig. 1:**
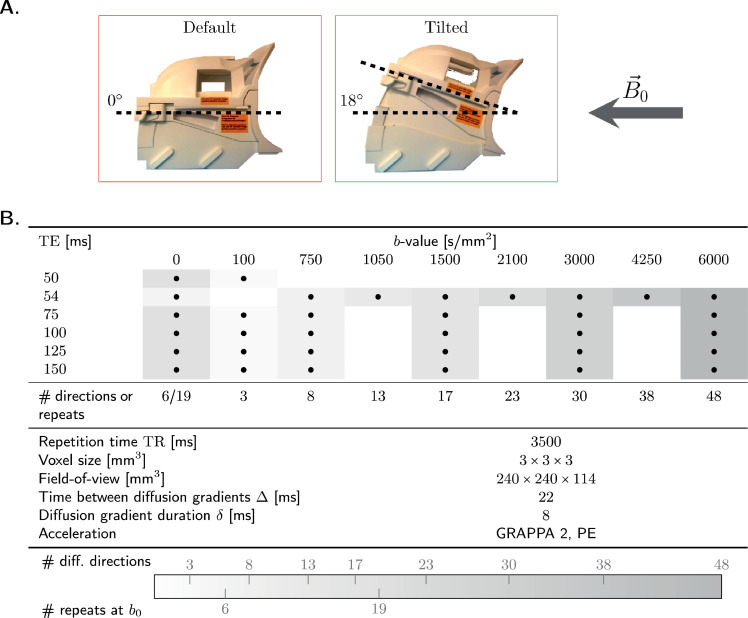
**A.** Data were acquired for each participant with the coil in default ($0^{\circ }$) and tilted ($18^{\circ }$) position with respect to the magnetic field ${\vec{B}}_{0}$. **B.** Acquisition parameters for the diffusion-$T_{2}$-correlation experiment: combinations of b-values and echo times TE used in this study are marked with a •-symbol. Number of diffusion directions or repetitions at $b_{0}$ is colour-coded for each b- and TE-value. The diffusion gradient duration δ and time between diffusion gradients Δ were kept fixed for all TE.

The study was approved by the Cardiff University School of Psychology Ethics Committee and written informed consent was obtained from the participants in the study. Five healthy volunteers (3 female, age range 25−31 y.o.) were scanned on a 3T 300 mT/m Connectom scanner equipped with a modified 20-channel head/neck tiltable coil (Siemens Healthineers, Erlangen, Germany). Qualitative preliminary observations of apparent $T_{2}$ orientation-dependence using this setup have previously been reported in Tax et al. (2020b). MRI data were acquired in the default ($0^{\circ }$) and tilted ($18^{\circ }$) orientations of the tiltable coil to ${\vec{B}}_{0}$ (Fig. 1A.) in two separate sessions. For each coil-orientation, the axial slices were aligned with the anterior commissure – posterior commissure line (AC-PC line). One of the subjects underwent a second scan in both the default and tilted head orientation to examine test-retest repeatability.

Diffusion-$T_{2}$ correlation data were acquired using a pulsed-gradient spin-echo echo-planar-imaging (PGSE-EPI) sequence (Stejskal and Tanner, 1965), with six different TEs to probe $T_{2},$ and nine b-values to probe diffusion, (Fig. 1B.). The timings of the diffusion encoding gradients were fixed for all echo times. The remaining parameters are reported in Fig. 1B. The acquisition was accelerated using the GRAPPA parallel imaging approach (Griswold et al., 2002) with acceleration factor of 2 in the phase encoding direction.

### 2.2 Data processing

#### 2.2.1 Pre-processing

The dMRI data were corrected for Rician noise bias (Koay, Ozarslan, Basser, 2009, St-Jean, Coupé, Descoteaux, 2016) using an estimate of the Gaussian noise standard deviation and the number of coils (Koay, Özarslan, Pierpaoli, 2009, St-Jean, De Luca, Tax, Viergever, Leemans, 2020) and an estimate of the signal (Cordero-Grande et al., 2019). The diffusion-$T_{2}$ data were checked for slice-wise outliers (Sairanen et al., 2018) and signal drift (Vos et al., 2016) and corrected for Gibbs ringing (Kellner et al., 2016), subject motion, eddy current-, susceptibility- and gradient nonlinearity induced geometrical distortions (Andersson, Skare, Ashburner, 2003, Andersson, Sotiropoulos, 2016, Glasser, Sotiropoulos, Wilson, Coalson, Fischl, Andersson, Xu, Jbabdi, Webster, Polimeni, Van Essen, Jenkinson, 2013) for each subject and each head orientation. The B-matrix was adjusted voxel-wise to account for the effects of gradient nonlinearities (Bammer, Markl, Barnett, Acar, Alley, Pelc, Glover, Moseley, 2003, Rudrapatna et al., 2021).

#### 2.2.2 Voxel-wise estimation

Parameter estimation was performed for each coil orientation (default ($0^{\circ }$) and tilted ($18^{\circ }$)) independently. Voxel-wise temporal signal-to-noise ratio (tSNR) estimates were obtained from the unprocessed $b=0\;\text{s}/{\text{mm}}^{2}$ images acquired at TE=54ms by dividing the mean of the b=0 images by their standard deviation.

On the pre-processed data, fibre orientation distribution functions (fODFs, Descoteaux, Deriche, Knosche, Anwander, 2008, Tournier, Calamante, Connelly, 2007) were estimated using multi-shell multi-tissue constrained spherical deconvolution (MSMT-CSD, Jeurissen et al., 2014) on the TE=54ms data. Peak orientations and magnitudes were subsequently extracted from the resulting fODFs. Voxels in which the majority of the fibres run primarily along one axis (single-fibre-population, SFP, voxels) were identified by imposing a relative threshold of 10% on the magnitude of the second-largest peak compared to the first peak (Tax et al., 2014) and an absolute threshold of 0.1, and a maximum CSF fraction of 1%. Supplementary Figure S1 shows SFP voxels from an example dataset. The angle θ of the SFP-orientations w.r.t. the main magnetic field ${\vec{B}}_{0}$ was then estimated (denoted by $\hat{\theta }$).

In SFP-voxels, the apparent $T_{2}$ was estimated from the $b=0\;\text{s}/{\text{mm}}^{2}$ signals by fitting a mono-exponential function of TE to the signal evolution (henceforth referred to as the ‘mono-exponential’ $T_{2}$ and denoted by $T_{2,\text{m}}$). Estimates of intra- and extra-axonal compartmental $T_{2}$ were obtained using all b-values and TEs as outlined in Appendix A.1. Briefly, the compartmental model of diffusion in WM describes the signal as a convolution of the signal associated with a population of perfectly parallel fibres with an fODF. Diffusion in the extra-axonal space is described by an axially symmetric ‘zeppelin’ tensor with two free parameters (parallel and perpendicular apparent diffusivity), and diffusion in the intra-axonal space by an axially symmetric ‘stick’ tensor with one free parameter (parallel apparent diffusivity, perpendicular apparent diffusivity is fixed to zero) (Jespersen, Kroenke, Østergaard, Ackerman, Yablonskiy, 2007, Kroenke, Ackerman, Yablonskiy, 2004). Each compartment has an associated apparent $T_{2}$ (Lampinen, Szczepankiewicz, Mårtensson, van Westen, Hansson, Westin, Nilsson, 2020, Tax, Rudrapatna, Witzel, Jones, 2017, Veraart, Novikov, Fieremans, 2018) denoted by $T_{2,\text{i}}$ and $T_{2,\text{e}}$ for the intra-axonal and extra-axonal transverse relaxation times, respectively. Both the mono-exponential and compartmental models were fitted using a nonlinear least-squares trust-region-reflective algorithm in Matlab (The Mathworks), with an implementation of the compartmental model as described by Lampinen et al. (2020) but with the possibility of inputting spatially varying b-matrices obtained from the gradient nonlinearity correction (Bammer, Markl, Barnett, Acar, Alley, Pelc, Glover, Moseley, 2003, Guo et al., 2020, Rudrapatna et al., 2021). The fit in each voxel was initialised three times within boundaries (which also served as constraints) $\left[0,\;0\;\mu {\text{m}}^{2}/\text{ms},\;0\;\mu {\text{m}}^{2}/\text{ms},\;0\;\mu {\text{m}}^{2}/\text{ms},\;30\;\text{ms},\;30\;\text{ms}\right]$ and $\left[1,\;3\;\mu {\text{m}}^{2}/\text{ms},\;3\;\mu {\text{m}}^{2}/\text{ms},\;2\;\mu {\text{m}}^{2}/\text{ms},\;300\;\text{ms},\;300\;\text{ms}\right]$ for f, $D_{∥,\text{i}},$ $D_{∥,\text{e}},$ $D_{⊥,\text{e}},$ $T_{2,\text{i}},$ and $T_{2,\text{e}},$ respectively. The solution with the lowest residual norm was taken for further analysis excluding voxels in which the estimates hit the fitting boundaries.

#### 2.2.3 Tractography

The estimated fODFs were used as input to perform probabilistic streamline tractography and automatic bundle segmentation using TractSeg (Wasserthal et al., 2018), resulting in 18 major WM bundles and their bilateral versions where applicable.

### 2.3 Data analysis

#### 2.3.1 Effect of fibre-orientation on $R_{2}=1/T_{2}$ estimates

The $T_{2}$ estimates were investigated as a function of fibre-orientation to ${\vec{B}}_{0}$ in SFP-voxels across the whole WM, along tracts and along tract-segments, pooled across subjects. The assignment of SFPs to tracts or tract-segments was obtained by a tractometry approach (Bells et al., 2011, Cousineau, Jodoin, Morency, Rozanski, Grand’Maison, Bedell, Descoteaux, 2017) in the native space of each head orientation. Briefly, a representative core-streamline was computed (Chamberland et al., 2018), and the bundles were subsequently subdivided into 20 segments along the core (Chamberland et al., 2019). SFPs were assigned to a segment of a tract if they were wholly contained within that tract segment and their orientation was within $15^{\circ }$ of the tangent to the core-streamline in that segment. Note that this tractometry approach achieves anatomical correspondence between the default and tilted coil-orientations (i.e., the tract segments) without the need for registration and interpolation to a common space.

The following analysis was performed in terms of transverse relaxation rate, $R_{2}=1/T_{2},$ instead of $T_{2}$ as this is more commonly used in the relaxation-anisotropy literature. $R_{2}$ can be represented as a function of fibre orientation θ to ${\vec{B}}_{0}$ as follows:

(1) $$R_{2}\left(\theta \right)=R_{2,\text{iso}}+f\left(\theta \right)\;,$$

(2) $$f\left(\theta \right)=R_{2,{\text{aniso}}_{1}}\cdot \sin^{2}\theta +R_{2,{\text{aniso}}_{2}}\cdot \sin^{4}\theta \;,$$

where $R_{2,\text{iso}}$ is the isotropic or orientation-independent component of $R_{2}\left(\theta \right)$ and f(θ) is the purely orientation-dependent or anisotropic component with $R_{2,{\text{aniso}}_{1}}$ and $R_{2,{\text{aniso}}_{2}}$ together reflecting the magnitude of $R_{2}$ dependence on θ. This is the most general representation used in studies investigating the orientation-dependence of the gradient-echo signal evolution and transverse relaxation rates (see Supplementary section 1). Other formulations either set $R_{2,{\text{aniso}}_{1}}$ and/or $R_{2,{\text{aniso}}_{2}}$ to 0 or to be linked. $R_{2,{\text{aniso}}_{1}}=0$ corresponds with representations chosen by Gil et al. (2016) and Knight et al. (2017) to describe the orientation-dependence of SE signals. $R_{2,{\text{aniso}}_{2}}=0$ has been used in Bender and Klose (2010); Lee et al. (2011), and $R_{2,{\text{aniso}}_{1}}=-1.5R_{2,{\text{aniso}}_{2}}$ typically represents magic angle effects (Birkl et al., 2020, Oh, Kim, Cho, Lee, 2013, Schyboll, Jaekel, Petruccione, Neeb, 2019). Finally, $R_{2,{\text{aniso}}_{1}}=R_{2,{\text{aniso}}_{2}}=0$ suggests a fully orientation-independent $R_{2}$. All variations of Eq. 2 are summarised in Table 1 and Appendix B. Tract-wise fitting was performed using function variations i)-iii) (Table 1) due to the limited range of fibre-angles θ in some of the tracts, but the most general ($R_{2,\text{iso}}\neq R_{2,{\text{aniso}}_{1}}\neq R_{2,{\text{aniso}}_{2}}\neq 0$) and the magic angle representations were also applied to the whole-brain WM data.

**Table 1:** Variations of Eq. 2 fitted to $R_{2}$ estimates in the whole brain white matter single fibre population voxels. Functions i)-iii) were also fitted to the SFP data tract-wise.

	Variation of Eq. 2	Whole brain WM	Individual tracts
i)	$R_{2}\left(\theta \right)=R_{2,\text{iso}}$	•	•
ii)	$R_{2}\left(\theta \right)=R_{2,\text{iso}}+R_{2,\text{aniso}}\cdot \sin^{2}\theta$	•	•
iii)	$R_{2}\left(\theta \right)=R_{2,\text{iso}}+R_{2,\text{aniso}}\cdot \sin^{4}\theta$	•	•
iv)	$R_{2}\left(\theta \right)=R_{2,\text{iso}}+R_{2,\text{aniso}}\cdot \left[1-\frac{1}{4}{\left(3\cos^{2}\theta -1\right)}^{2}\right]$	•	
v)	$R_{2}\left(\theta \right)=R_{2,\text{iso}}+R_{2,{\text{aniso}}_{1}}\cdot \sin^{2}\theta +R_{2,{\text{aniso}}_{2}}\cdot \sin^{4}\theta$	•

**Table B1:** Summary of the relevant trigonometric relations.

a+b·cos(2θ)	$A+B\cdot \sin^{2}\theta$	A=a+b;B=−2b
$a+b\cdot \cos\left(2\theta \right)-\frac{1}{4}b\cdot \cos\left(4\theta \right)$	$A+B\cdot \sin^{4}\theta$	$A=a+\frac{3}{4}b;\;B=-2b$
$a+b\cdot {\left(3\cos^{2}\theta -1\right)}^{2}$	$A+B\cdot \sin^{2}\theta -1.5B\cdot \sin^{4}\theta$	A=a+4b;B=−6b
a+bcos(2θ)+ccos(4θ)	$A+B\cdot \sin^{2}\theta +C\cdot \sin^{4}\theta$	A=a+b+c;B=−2b−8c;C=8c

To test for the most parsimonious representation of the signal, Akaike’s Information Criterion (AIC) was used (Akaike, 1974):

(3) AIC=2·K+N·ln(RSS/N),

where K is the number of fitting parameters, N is the number of data points and RSS is the residual sum of squares. To compare the relative performance of representations considered, the AIC-values were rescaled:

(4) $$\Delta_{\text{AIC}}=\text{AIC}-{\text{AIC}}_{\text{min}}\;,$$

where ${\text{AIC}}_{\text{min}}$ is the minimum of the different AIC values in the set. Per Burnham and Anderson, 2004, $\Delta_{\text{AIC}}$ values allow comparison of the relative merits of representations in the set as follows: representations having $\Delta_{\text{AIC}}\leq 2$ are considered to have similar substantial support as the representation with ${\text{AIC}}_{\text{min}},$ those with $4\leq \Delta_{\text{AIC}}\leq 7$ have considerably less evidence, and those with $\Delta_{\text{AIC}}\geq 10$ have no support. Moreover, per Arnold (2010), anisotropic models were discarded from the AIC comparison for which the 85% confidence interval of ${\hat{R}}_{2,\text{aniso}}$ included zero.

Estimates for the parameters in Eq. 2 – which will be indicated as such by a hat ^ – were obtained to: 1) study overall anisotropy effects by pooling all SFP voxels within a pre-defined region of interest (ROI) (i.e. a WM mask or bundle reconstructed via tractography) for both the default and tilted coil-orientation; and 2) analyse whether there was a significant effect of head orientation on $R_{2}$ using the tiltable coil by taking a segment-wise approach, where the tract-segments derived from tractometry establish the spatial correspondence between coil-orientations. The second (segment-wise) approach aims to compare the same anatomical region in the tilted and non-tilted orientation as opposed to relying on the natural twists and turns of anatomical structures within the brain; the first approach does not disentangle these two sources of reorientation and pools all the voxels from different tilts and different structures. Here, endpoint-segments (20% on each side of a tract) and segments with fewer than 3 voxels were excluded to minimise the effects of noise and fanning. By assuming that the orientation-independent component $R_{2,\text{iso}}$ is the same for the default- and tilted coil-orientation, estimates ${\hat{R}}_{2,\text{aniso}}^{\text{s}}$ – where superscript s is a reminder that this was done segment-wise – could be obtained by plotting ${\left[{\hat{R}}_{2}^{\text{s}}\right]}_{0^{\circ }}-{\left[{\hat{R}}_{2}^{\text{s}}\right]}_{18^{\circ }}$ as a function of $\sin^{2}\left({\hat{\theta }}_{0^{\circ }}^{\text{s}}\right)-\sin^{2}\left({\hat{\theta }}_{18^{\circ }}^{\text{s}}\right)$ or $\sin^{4}\left({\hat{\theta }}_{0^{\circ }}^{\text{s}}\right)-\sin^{4}\left({\hat{\theta }}_{18^{\circ }}^{\text{s}}\right)$ respectively. Here, ${\hat{R}}_{2}^{\text{s}}$ and ${\hat{\theta }}^{\text{s}}$ are segment-wise estimates obtained by taking the mean ${\hat{R}}_{2}$ and mean orientation, i.e. first eigenvector of the scatter matrix (Basser, Pajevic, 1999, Mardia, Jupp, 2009, Tax et al., 2015), across SFPs in a segment. A linear fit can then be performed through all segment-wise estimates in an ROI (i.e., WM mask or tract).

#### 2.3.2 Repeatability of the experiment

For comparison of the test-retest data, an intraclass correlation coefficient (ICC) across segments was calculated as follows:

(5) $$\begin{array}{cc} & \text{ICC}=\frac{1}{2Ns^{2}}\sum_{n=1}^{N}\left(x_{0\text{°},n}-\bar{x}\right)\left(x_{18\text{°},n}-\bar{x}\right)\\ & \text{with}\;\bar{x}=\sum_{n}\left(x_{0\text{°},n}+x_{18\text{°},n}\right)/2N\\ & \text{and}\;s^{2}=\sum_{n}\left({\left(x_{0\text{°},n}-\bar{x}\right)}^{2}+{\left(x_{18\text{°},n}-\bar{x}\right)}^{2}\right)/2N\;, \end{array}$$

where N is the total number of data points for each head orientation (equivalent to number of segments multiplied by the number of tracts), and $x_{0^{\circ }/18^{\circ },n}$ are the corresponding ${\hat{R}}_{2}$ or $\hat{\theta }$ estimates for either head orientation.

## 3 Results

**Fig. 2:**
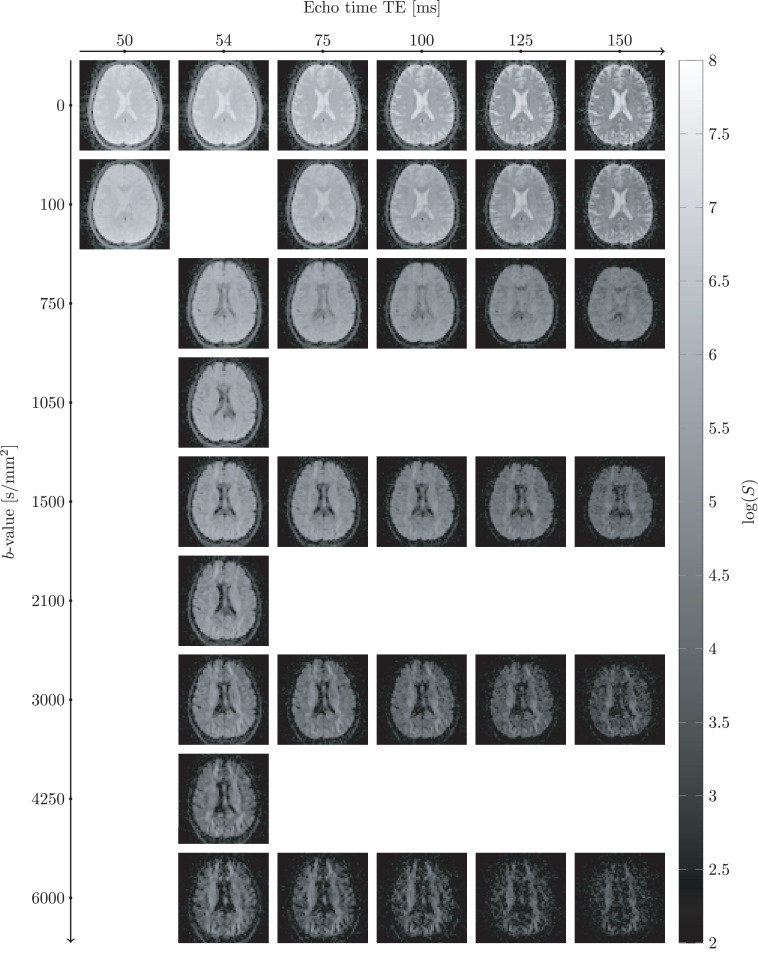
The diffusion-$T_{2}$ correlation data were acquired by simultaneously varying b-value and the echo time TE in a diffusion-weighted spin-echo EPI sequence. The example data were acquired in default head orientation with diffusion gradients aligned with the superior-inferior axis.

Fig. 2 shows images of the diffusion–relaxation correlation dataset for different b-values and TEs in a single subject, in the default coil-orientation. Visual comparison with images from the tilted coil-orientation (Supplementary Figure S2) did not reveal major differences in image-quality (such as signal dropouts or other artifacts), indicating that data acquisition in the tilted orientation could be performed reliably. This was further corroborated by feedback from the participants, who did not report major discomfort in the tilted coil-orientation.

### 3.1 Signal-to-noise ratio

**Fig. 3:**
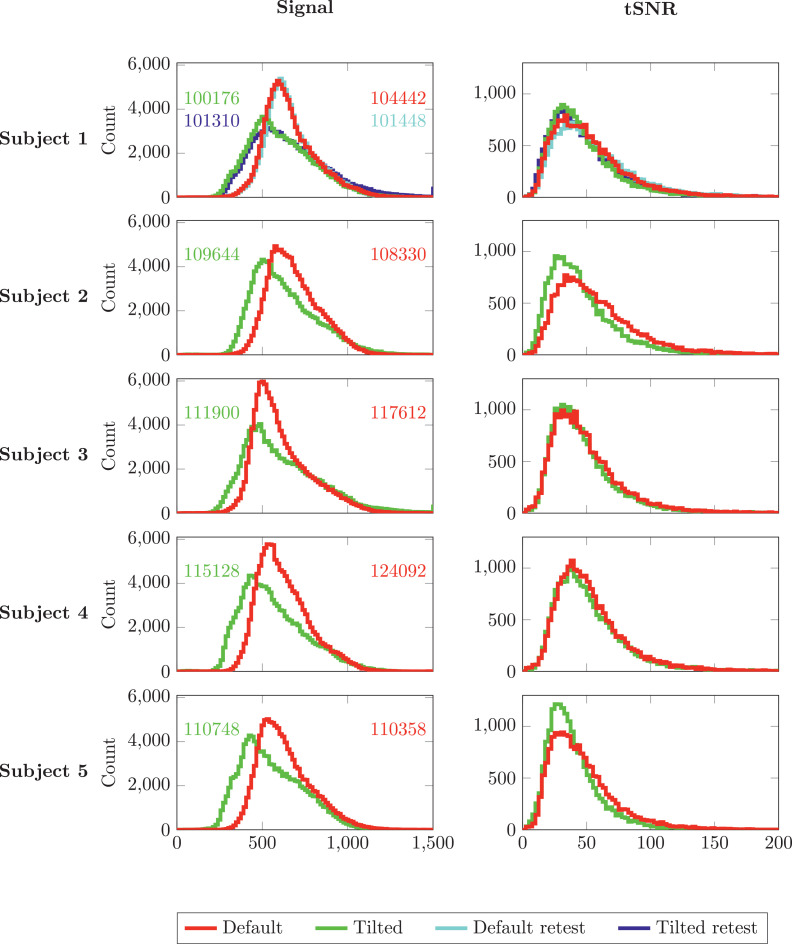
Distributions of the signal (left column) and temporal SNR (tSNR, right column) in white matter from the data acquired at $b=0\;\text{s}/{\text{mm}}^{2}$ and TE=54ms in all subjects (rows) for two receive-coil orientations: default in red and tilted in green. For the first subject, default retest (cyan) and tilted retest (blue) signal- and SNR distributions are also shown. Total number of WM voxels for each subject and each head orientation are noted next to the corresponding distribution in the left column.

Fig. 3 displays histograms of the signal and tSNR in a WM mask for different subjects in the default and tilted coil-orientations. While the signal-histograms reveal an overall lower signal in the tilted orientation, the tSNR-histograms suggest that the tSNR remains relatively unchanged when tilting the coil, and when performing retest experiments.

### 3.2 Effect of fibre-orientation on $R_{2}=1/T_{2}$ estimates

#### 3.2.1 Single-fibre populations across the WM

**Fig. 4:**
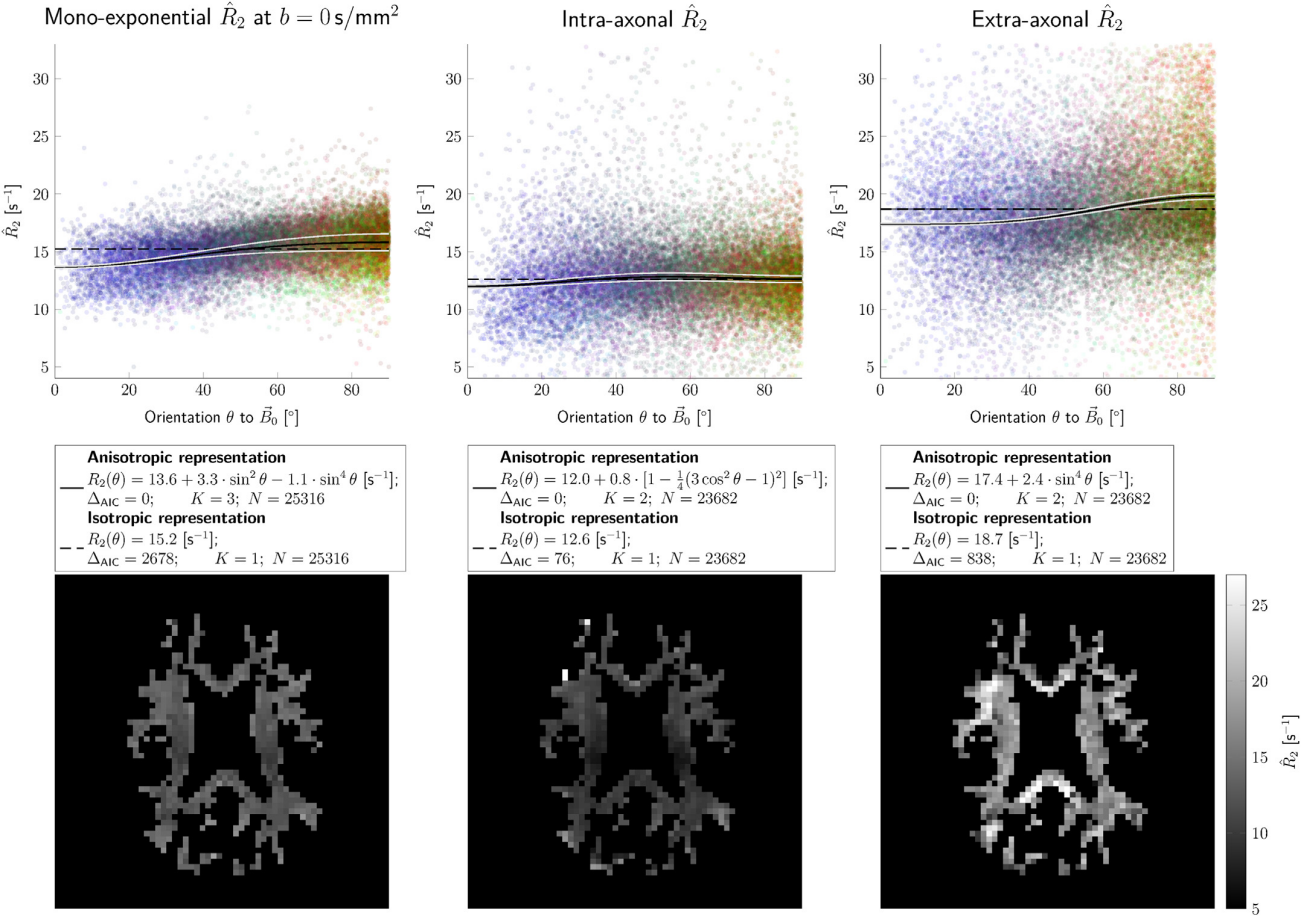
Mono-exponential, intra- and extra-axonal relaxation parameter estimates in SFP voxels across the white matter (columns from left to right, respectively). The top row shows ${\hat{R}}_{2}$-values from all subjects in both head orientations plotted against fibre orientation $\hat{\theta }$ to ${\vec{B}}_{0}$. Colours represent fibre orientations in scanner coordinates (red,blue and green stand for left-right (LR), superior-inferior (SI) and anterior-posterior (AP), respectively). The black solid lines represent the best fitting curves, while the dashed lines indicate the isotropic case (i.e. $R_{2,{\text{aniso}}_{1}}=R_{2,{\text{aniso}}_{2}}=0$ in Eq. 2). The white lines outline 95% confidence intervals. The best fitting functions, $\Delta_{\text{AIC}},$ number of fitting parameters K, and number of fitting points N, are displayed in the legend. The bottom row shows examples of corresponding ${\hat{R}}_{2}$-maps in WM from a single subject in the default head orientation.

Fig. 4 shows the estimated $R_{2}$-values as a function of estimated fibre-angle w.r.t. ${\vec{B}}_{0}$ for SFPs across the WM for both coil-orientations and all subjects. Both mono-exponential estimates ${\hat{R}}_{2,\text{m}}=1/{\hat{T}}_{2,\text{m}}$ as well as compartmental intra- and extra-axonal estimates (${\hat{R}}_{2,\text{i}}$ and ${\hat{R}}_{2,\text{e}}$) are shown, along with the best-fitting representation from Table 1 (solid black line and confidence interval boundaries in white). The best-fitting representation for each scenario was selected based on their AIC-values Eqs. 3 and (4) by choosing from the subset of representations with $\Delta_{\text{AIC}}\leq 2$ – where the representation with the minimum AIC has $\Delta_{\text{AIC}}=0$ per definition – the one with the lowest number of fitting parameters. Dashed black lines in the same plot indicate the isotropic case $R_{2}\left(\theta \right)=R_{2,\text{iso}}$. All fitting parameters resulting from the variations of Eq. 2 can be found in Supplementary Table S1 with corresponding curves shown in Supplementary Figure S3. Supplementary Figure S4 also shows $R_{2}$ as a function of fibre orientation to ${\vec{B}}_{0}$ for those SFP voxels with ‘orientational coherence’ parameter $p_{2}$ above 0.5 (Lampinen, Szczepankiewicz, Mårtensson, van Westen, Hansson, Westin, Nilsson, 2020, Novikov, Veraart, Jelescu, Fieremans, 2018), see also Appendix A.1.1. Corresponding $T_{2}$-maps are shown in Supplementary Figure S5.

${\hat{R}}_{2,\text{e}}$ is generally higher than ${\hat{R}}_{2,\text{i}}$. Directional dependence can be observed in all ${\hat{R}}_{2}$ estimates (most visibly in the mono-exponential and extra-axonal ${\hat{R}}_{2}$), with AIC-values suggesting that the fit is improved when the anisotropic coefficients $R_{2,{\text{aniso}}_{1}}$ and/or $R_{2,{\text{aniso}}_{2}}$ were not equal to 0. In the mono-exponential case, ${\hat{R}}_{2,\text{m}}$ was best represented by the most general expression $R_{2}\left(\theta \right)=13.6+3.3\cdot \sin^{2}\theta -1.1\cdot \sin^{4}\theta$. For the compartmental cases, ${\hat{R}}_{2,\text{i}}$ was described best by the representation $R_{2}\left(\theta \right)=12.0+0.8\cdot \left[1-\frac{1}{4}{\left(3\cos^{2}\theta -1\right)}^{2}\right],$ while the preferred representation for ${\hat{R}}_{2,\text{e}}$ was $R_{2}\left(\theta \right)=17.4+2.4\cdot \sin^{4}\theta$.

#### 3.2.2 Single-fibre populations along different tracts

**Fig. 5:**
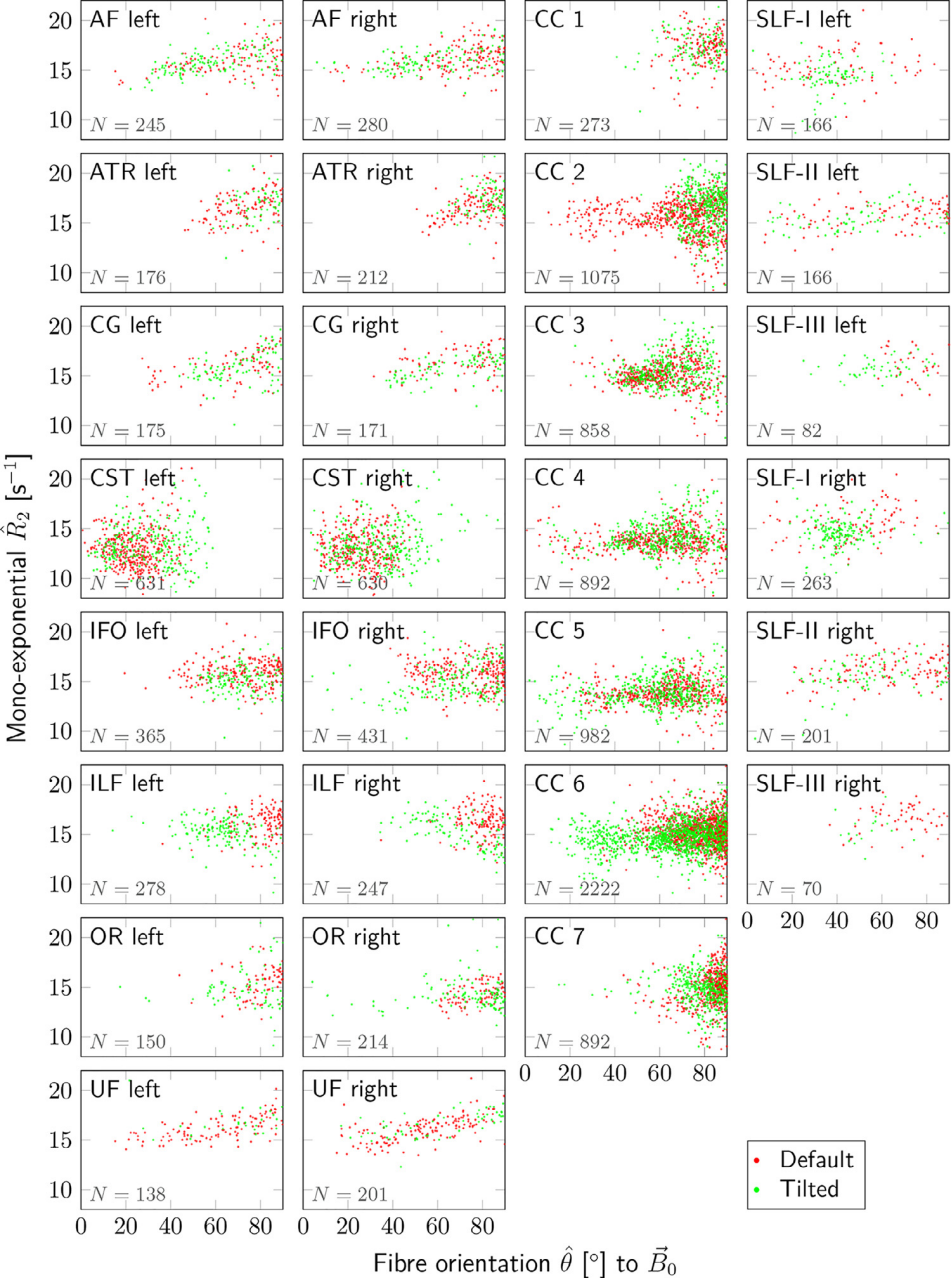
Mono-exponential relaxation rates ${\hat{R}}_{2}$ estimated from data acquired at $b_{0}$ are plotted against fibre orientation $\hat{\theta }$ to the magnetic field ${\vec{B}}_{0}$ for default (red) and tilted (green) head orientations. Each point represents one of the SFP voxels from one of 29 fibre tracts (separate plots, fibre tract name in top left corner) in each subject. Total number, N, of voxels included from all subjects and orientations for each tract is indicated in bottom left corner of each plot. Evidently, adding an acquisition in the tilted position enables the exploration of a wider range of angles θ compared to the default position along various tracts.

**Fig. 6:**
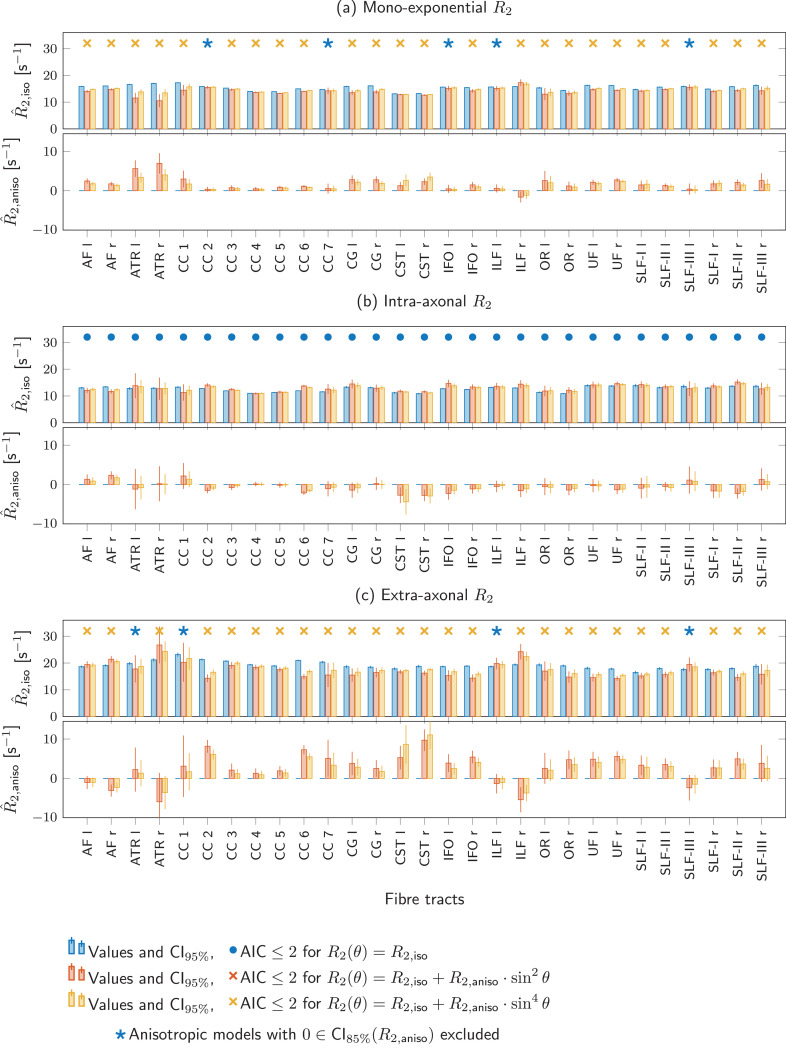
Isotropic and anisotropic components of (a) mono-exponential, (b) intra-axonal, or (c) extra-axonal $R_{2}\left(\theta \right)$ were simultaneously estimated from default and tilted data from all subjects for each fibre tract using $R_{2}\left(\theta \right)=R_{2,\text{iso}}+f\left(\theta \right),$ where f(θ) was i) 0 (blue •); ii) $R_{2,\text{aniso}}\cdot \sin^{2}\theta$ (red ×); and iii) $R_{2,\text{aniso}}\cdot \sin^{4}\theta$ (yellow ×). Top and bottom plots show bar plots and 95% confidence bounds of the isotropic component $R_{2,\text{iso}}$ and the magnitude of anisotropy $R_{2,\text{aniso}},$ respectively. Symbols •, and × above grouped bar-plots for each fibre tract indicate which model outperformed the others (lowest AIC), and symbol ★ highlights those tracts for which the 85% confidence interval of $R_{2,\text{aniso}}$ included 0 for both anisotropic models.

Fig. 5 plots ${\hat{R}}_{2,\text{m}}$ as a function of $\hat{\theta }$ for SFPs along different tracts for all subjects and both coil orientations (similar plots for the intra- and extra-axonal $R_{2}$-estimates are shown in Supplementary Figure S6). Parameter estimates for different variations of Eq. 2 (listed in Table 1) per tract are visualised in Fig. 6.

The extra-axonal ${\hat{R}}_{2,\text{iso}}$ varies more across tracts than the mono-exponential and intra-axonal ${\hat{R}}_{2,\text{iso}}$. This variability is most pronounced in ${\hat{R}}_{2,\text{iso}}$ values from the anisotropic models; extra-axonal ${\hat{R}}_{2,\text{iso}}$ from the isotropic model vary less across tracts. Concomitantly, the magnitude of the extra-axonal ${\hat{R}}_{2,\text{aniso}}$ is overall larger than the intra-axonal ${\hat{R}}_{2,\text{aniso}}$. A comparison of AICs (with the lowest AIC indicated by the colour of the symbol, × for anisotropic-model preference and • or ★ for isotropic) reveals that in all tracts the isotropic model is preferred for the intra-axonal ${\hat{R}}_{2}$. For the mono-exponential and extra-axonal ${\hat{R}}_{2},$ an anisotropic model is preferred over the isotropic model in 24/29 and 25/29 of the tracts respectively, and in the remaining scenarios anisotropic models were excluded from the AIC comparison as the 85% confidence intervals of ${\hat{R}}_{2,\text{aniso}}$-values included 0, ultimately leading to the selection of the isotropic model (marked with a ★). The estimates and confidence intervals for these scenarios are listed in Supplementary Tables S2 and S3. For the tracts for which an anisotropic model was preferred in either or both hemispheres, only the left/right ILF had ${\hat{R}}_{2,\text{aniso}}$ estimates outside each others’ confidence interval for the mono-exponential $R_{2},$ and the left/right CST and SLF-III for the extra-axonal $R_{2}$.

#### 3.2.3 Effect of coil-orientation

As seen in Fig. 5, adding an acquisition in the tilted position (green points) enables the exploration of a wider range of angles compared to the default position only (red points) along various tracts. Most notably, these include CC2, CC6, CST, ILF, and various parts of the SLF.

**Fig. 7:**
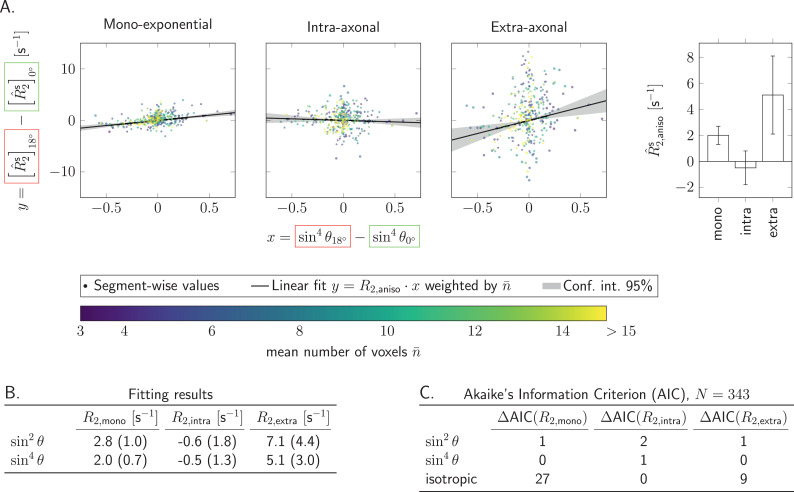
Relaxation anisotropy was probed segment-wise by comparing values estimated in the default and tilted coil-orientations in segments 5 to 16 and with at least 3 voxels per segment. A. Differences between per-segment ${\hat{R}}_{2}^{\text{s}}\left(\theta \right)$-values $y={\left[{\hat{R}}_{2}^{\text{s}}\right]}_{0^{\circ }}-{\left[{\hat{R}}_{2}^{\text{s}}\right]}_{18^{\circ }}$ were plotted against differences in corresponding $\sin^{4}\theta$-values for mono-exponential (estimated at $b=0\;\text{s}/{\text{mm}}^{2}$), intra- and extra-axonal $R_{2}$-values. Colours correspond to the number of voxels $\bar{n}$ per segment, averaged between the default and tilted head positions. The magnitude of anisotropy ${\hat{R}}_{2,\text{aniso}}^{\text{s}}$ was estimated from the linear fit $y={\hat{R}}_{2,\text{aniso}}^{\text{s}}\cdot x,$ where $x=\sin^{4}{\hat{\theta }}_{0^{\circ }}^{\text{s}}-\sin^{4}{\hat{\theta }}_{18^{\circ }}^{\text{s}}$. The resulting ${\hat{R}}_{2,\text{aniso}}^{\text{s}}$-values are shown in the bar plot on the right hand side, along with the corresponding 95% confidence intervals. The tables list fitting results for anisotropic representations with $\sin^{2}\theta$ and $\sin^{4}\theta$ terms (B.), and the effective AIC values ($\Delta_{\text{AIC}}=\text{AIC}-{\text{AIC}}_{\text{min}}$) for each representation including isotropic x=0 for mono-exponential, intra- and extra-axonal ${\hat{R}}_{2}$-values (C.). Number of data points included in the fitting was N=343.

The effect of coil-reorientation on mono-exponential, intra- and extra-axonal ${\hat{R}}_{2}$-estimates in the WM is further explored in Fig. 7. In contrast to the previous results, here we explore *segment-wise* differences between coil-orientations. Segment-wise ${\hat{R}}_{2}^{\text{s}}$-differences between default and tilted head orientations are plotted against differences in $\sin^{4}{\hat{\theta }}^{\text{s}}$ in the WM for all subjects. For each case, ${\hat{R}}_{2,\text{aniso}}^{\text{s}}$ was estimated from a linear fit through the origin, the values and their confidence intervals are summarised in the box plots. Corresponding $\Delta_{\text{AIC}}$ values are shown in the table underneath the plots. For comparison, AIC under the assumption that there is no angular dependence were also estimated and included in the table. For mono-exponential and extra-axonal ${\hat{R}}_{2}$ values the anisotropy was better described by a $\sin^{4}\theta$-dependence (lowest AIC values), while orientational dependence of the intra-axonal ${\hat{R}}_{2}$ was equally well represented by an isotropic and $\sin^{4}\theta$-dependence. In supplementary Figures S7 and S8 we also provided segment-wise analysis under the reduced influence of fibre-orientational dispersion (by including only those SFP voxels with $p_{2}>0.5$ and a stricter angular threshold when calculating segment-wise $R_{2}$- and θ-values). Our results show that the orientational anisotropy effects were stronger in the extra-axonal compartment, and weaker in the intra-axonal compartment. Mono-exponential ${\hat{R}}_{2,\text{aniso}}$-values lie between those values, as expected.

### 3.3 Test-retest reliability of results

**Fig. 8:**
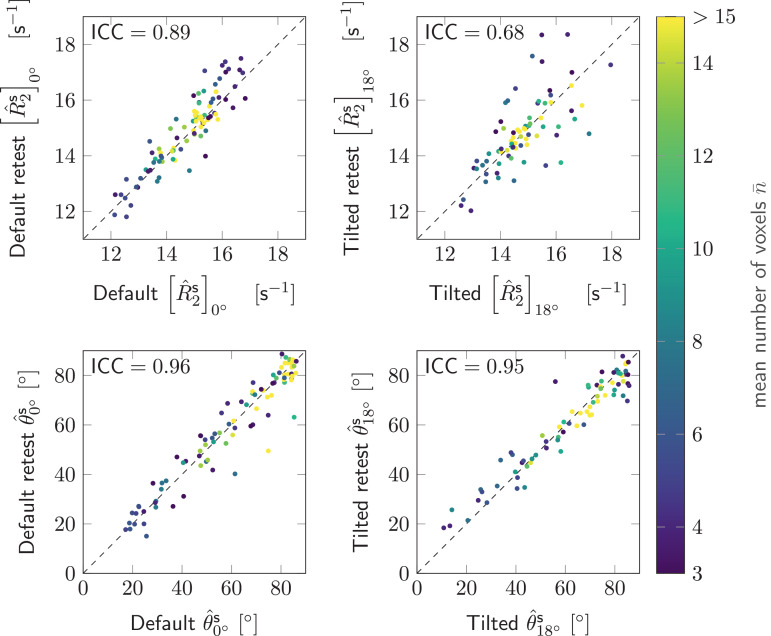
Repeatibility of estimates was investigated from the test-retest data acquired in one subject in default (left column) and tilted (right column) coil-orientations. Each data point represents a single tract segment with its test value along the horizontal axis and retest value along the vertical axis. The test and retest estimates are plotted for ${\hat{R}}_{2,\text{m}}^{\text{s}}$ (top row) and the fibre orientation ${\hat{\theta }}^{\text{s}}$ to ${\vec{B}}_{0}$ (bottom row). The colours correspond to the number of voxels per segment, averaged between the default and tilted head positions. Intraclass correlation coefficients (ICC) are included in the top left corner of each plot.

Test-retest segment-wise mono-exponential ${\hat{R}}_{2,\text{m}}^{\text{s}}$ and ${\hat{\theta }}^{\text{s}}$ estimates from a single participant are plotted in Fig. 8. Generally, the reproducibility of ${\hat{\theta }}^{\text{s}}$ were excellent (0.9<ICC) and the reproducibility of ${\hat{R}}_{2,\text{m}}^{\text{s}}$ were good (0.75<ICC≤0.9) to moderate (0.5<ICC≤0.75) for both head orientations (Koo and Li, 2016). ICC values were slightly higher in default head position, as compared to the tilted head position.

## 4 Discussion

### 4.1 Summary of results and comparison to previous studies

In this work we have investigated the mono-exponential and compartment-specific $T_{2}$-anisotropy in white matter using a tiltable receive coil and ultra-strong diffusion gradients at 3T. Typical $T_{2}$-anisotropy measurements involve re-orienting the head inside the receive coil, using additional padding to stabilise the head and achieve maximum re-orientation (Bender, Klose, 2010, Knight, Wood, Couthard, Kauppinen, 2015). We have shown that a tiltable coil can help ameliorating expected challenges associated with such experiments, i.e. unintended SNR variations across different head orientations caused by differences in proximities to the receiver coil, and increased susceptibility to motion and artefacts due to patient discomfort. In addition, whereas the majority of previous works have acquired separate diffusion- (PGSE) and $T_{2}$- (CPMG) weighted scans, we varied b-value and TE simultaneously in a diffusion–relaxation correlation experiment (PGSE) which obviates registration between modalities. Compared to previous works disentangling compartmental $T_{2}$ (McKinnon, Jensen, 2019, Veraart, Novikov, Fieremans, 2018), we fixed the diffusion encoding timings in order to avoid including time-dependent effects which would confound each variation of TE with additional variations of encoding timings, and did not adopt the computation of rotational invariants from ‘shells’ with a unique b- and TE combination (Appendix A) to enable incorporation of spatiotemporally varying b-matrices (Henriques et al., 2019). To investigate $R_{2}$ in SFP-voxels along tracts and segment-wise, spatial correspondence between different coil-orientations was obtained by a tractometry approach in the native space, thereby reducing potential confounds from misregistration. The joint acquisition allowed the separation of compartmental anisotropy effects: myelin water likely had a minor contribution to the signal decay because of the relatively long TE while contributions from intra- and extra-axonal water could be disentangled based on differences in their diffusion anisotropy. Previous works have mostly focused on the mono-exponential $T_{2}$ orientation-dependence or have separated compartmental orientation-dependence based on multi-component modelling of the signal (e.g. a complex three-pool model of the multi-echo GRE signal in Sati et al. (2013)). Introducing diffusion-weighting as done in this work allows for an independent orthogonal dimension to better separate compartmental contributions and orientation-dependence (de Almeida Martins, Tax, Szczepankiewicz, Jones, Westin, Topgaard, 2020, Kleban et al., 2020, Tax, Rudrapatna, Witzel, Jones, 2017, Veraart, Novikov, Fieremans, 2018). Simulations for varying noise levels (Supplementary Figure S10), intra- and extra-axonal $R_{2}$ (Supplementary Figure S11), and extra-axonal $R_{2,\text{aniso}}$ (Supplementary Figure S12) show that the method can indeed disentangle differences in compartmental $R_{2}$ and detect $R_{2}$-orientation-dependence. Furthermore, whereas most studies use DT-based estimates of fibre orientation (first eigenvector) and fibre alignment (FA or linear shape coefficient), we aimed to reduce confounding effects from multiple crossing fibre populations in a voxel by using estimation techniques beyond DTI (Jeurissen, Tournier, Dhollander, Connelly, Sijbers, 2014, Tax et al., 2014). Altogether, the adoption of these recent advances in acquisition and processing should have improved the robustness of the analysis.

We first explored fibre-orientation dependence of the spin-echo signal evolution across brain WM by assessing the apparent relaxation rate (estimated by fitting a mono-exponential function to the spin-echo signal at b=0) in each voxel as a function of the fibre orientation to ${\vec{B}}_{0}$ in that voxel. The relation between $R_{2}$ and θ estimates pooled from all subjects and both coil orientations was best represented by ${\hat{R}}_{2}\left(\theta \right)=13.6+3.3\cdot \sin^{2}\theta -1.1\cdot \sin^{4}\theta \;{\text{s}}^{-1}$ (based on the lowest AIC). The difference between the absolute minimum and maximum of ${\hat{R}}_{2}\left(\theta \right)$ can be interpreted as a magnitude of anisotropy, which in this case is $2.2\;\left[1.5,2.8\right]\;{\text{s}}^{-1}$. The magnitude of $R_{2}$-anisotropy observed in this work is consistent with previously reported values at 3T of $0\cdots 1.5\;{\text{s}}^{-1}$ (Gil et al., 2016), and $1.5\cdots 1.8\;{\text{s}}^{-1}$ (Knight et al., 2017, where the magnitude of anisotropy was estimated using their Figure 3EF and Equation 15). Notably, our tract-wise estimates of $R_{2,\text{m}}$ anisotropy are comparable to those obtained by Gil et al. (2016). The limited angle range w.r.t. ${\vec{B}}_{0}$ in some fibre tracts may have disadvantaged tract-wise estimation, potentially explaining the few occurrences of negative ${\hat{R}}_{2}$ in the right ILF for $R_{2,\text{m}}$ and $R_{2,\text{e}},$ and the AF and right ATR for $R_{2,\text{e}}$ based on the 85% confidence interval (Tables S2 and S3). Additionally, we studied the effect of head re-orientation relative to ${\vec{B}}_{0}$ by comparing the $R_{2}$ and θ averages over each fibre-tract-segment obtained in default coil alignment to those estimated when the coil was tilted at $18^{\circ },$ which led to a similar magnitude of anisotropy of $2.1\;\left[1.7,2.4\right]\;{\text{s}}^{-1}$.

By employing diffusion–relaxation correlation acquisitions and ultra-strong gradients we were able to disentangle intra- and extra-axonal SE signals, with our results suggesting slower intra-axonal signal decay (${\hat{T}}_{2,\text{i}}=89\;\left[60,118\right]\;\text{ms}$ and 83[62,102]ms for fibre tracts oriented at $0^{\circ }\cdots 30^{\circ }$ and $70^{\circ }\cdots 90^{\circ }$ to ${\vec{B}}_{0},$ respectively) compared to extra-axonal signal decay (${\hat{T}}_{2,\text{e}}=58\;\left[29,87\right]\;\text{ms}$ and 49[28,71]ms for fibre tracts oriented at $0^{\circ }\cdots 30^{\circ }$ and $70^{\circ }\cdots 90^{\circ }$ to ${\vec{B}}_{0},$ respectively), which are consistent with previously reported values:

• Intra-axonal $T_{2}=80\;\text{ms}$ and extra-axonal $T_{2}=65\;\text{ms}$ Tax et al. (2017).
• Intra-axonal $T_{2}=70\cdots 110\;\text{ms}$ and extra-axonal $T_{2}=50\cdots 65\;\text{ms}$ Veraart et al. (2018).
• Intra-axonal $T_{2}=69\cdots 107\;\text{ms}$ and extra-axonal $T_{2}=60\cdots 68\;\text{ms}$ Lampinen et al. (2020).
• Intra-axonal $T_{2}=50\cdots 110\;\text{ms}$ and extra-axonal $T_{2}=40\cdots 70\;\text{ms}$ McKinnon and Jensen (2019).

McKinnon and Jensen (2019) additionally observed orientational anisotropy in the intra-axonal space. We estimated that their magnitude of anisotropy was $2.7\;{\text{s}}^{-1}$ by fitting a $\sin^{2}\theta$-representation to the $1/T_{2}$-values from their Fig. 8, which was larger than the estimates obtained in our study. We have also applied their approach to our data and compared the resulting values to those obtained in this work in Supplementary Fig. S9. We obtained similar intra-axonal ${\hat{R}}_{2,\text{iso}}$ ($11.3\;\left[11.2,11.4\right]\;{\text{s}}^{-1}$ vs $12.0\;\left[11.8,12.1\right]\;{\text{s}}^{-1}$) and ${\hat{R}}_{2,\text{aniso}}$ ($1.0\;\left[0.5,2.4\right]\;{\text{s}}^{-1}$ vs $0.8\;\left[0.6,1.0\right]\;{\text{s}}^{-1}$) from our data using the two methods. The difference between this work and the reported results of McKinnon and Jensen (2019) may be due to a difference in selection of SFP voxels (from fODFs vs DT-MRI), the fitting (to individual voxel values vs a graphical fit to the mean in their paper), and fixed vs variable diffusion times with changing TE, among others. For the sake of completeness we also report the extra-axonal ${\hat{R}}_{2}$ estimated per Eq [3] in McKinnon and Jensen (2019), confirming the larger magnitude of $R_{2,\text{aniso}}$ in the extra-axonal space. However, this approach requires fixing $D_{\text{i}}$ and its accuracy can affect the accuracy of ${\hat{R}}_{2,\text{e}},$ which puts this approach “on a less firm foundation” (McKinnon and Jensen, 2019) than the approach for estimating ${\hat{R}}_{2,\text{i}},$ potentially further explaining the observed difference in extra-axonal ${\hat{R}}_{2,\text{iso}}$. We refer to Supplementary Table S4 for a summary of our values compared to previous studies.

### 4.2 Mechanisms for $R_{2}=1/T_{2}$ orientation dependence

Studies exploring the mechanisms underlying $T_{2}^{*}$ and $T_{2}$-orientation dependence have been performed for various field strengths (3T, 7T, and 9.4T), TE-ranges, and experimental conditions (*in* and *ex vivo*, different temperatures and species). The majority of studies suggest a primary role for myelin susceptibility effects, potentially combined with susceptibility from vasculature and magic-angle effects.

In this study we observed that the mono-exponential ${\hat{R}}_{2,\text{m}}\left(\theta \right)$-behaviour was best described by a superposition of $\sin^{2}\theta$ and $\sin^{4}\theta$ terms. Previous studies have attributed this to anisotropic susceptibility effects (Lee, van Gelderen, Kuo, Merkle, Silva, Duyn, 2011, Oh, Kim, Cho, Lee, 2013), contributions from myelin water (Wharton and Bowtell, 2013), interactions with deliberately applied gradients (Knight et al., 2017), and combinations of these sources with each other and with magic angle effects (Oh et al., 2013). Contributions from myelin water are likely small here, as at the shortest TE of 54ms used in this work the direct myelin water signal can be considered negligible. However, simulation studies (Brusini, Menegaz, Nilsson, 2019, Harkins et al., 2016) have shown that myelin water has the potential to subtly influence dMRI results and bias models if neglected, e.g. through exchange. This has yet to be further validated in *in vivo* human measurements. Furthermore, for the estimation of ${\hat{R}}_{2,\text{m}}$ no deliberate field gradients were applied.

Disentangling contributions from different compartments may further elucidate the origins of $T_{2}$-orientation dependence. The intra-axonal ${\hat{R}}_{2,\text{i}}\left(\theta \right)$ demonstrated a subtle combined $\sin^{2}\theta$ and $\sin^{4}\theta$-dependence in SFPs across the whole WM with $\Delta_{\text{AIC}}=1$ (Supplementary Table S1, Figure S3), which hints at a combination of mechanisms that shorten the coherence lifetime and has to be further investigated. Susceptibility effects could play a role as axons are no perfect cylinders and the architecture of myelin is not homogeneous (Sukstanskii and Yablonskiy, 2014), as well as potential differences in axon size of tracts running in different directions. Magic angle effects in nervous tissue have mostly been attributed to dipole-dipole interactions of bound water protons to collagen (Chappell et al., 2004), myelin (Lee, van Gelderen, Kuo, Merkle, Silva, Duyn, 2011, Schyboll, Jaekel, Petruccione, Neeb, 2019), and microtubule and neurofilaments (Birkl et al., 2020). In fact, the magic angle representation was found to have substantial support in our data (Fig. 4). Henkelman et al. (1994) did not observe $T_{2}$ magic angle effects in WM on 1.5T and note that in tube-like structures such as myelinated axons, water molecules can bind to the surface (in this case the phospholipid bilayer), but as this has no preferred direction water molecules can be oriented along different axes than the cylinder-axis. They therefore consider relaxation-anisotropy effects through this mechanism unlikely. Schyboll et al. (2020) suggest that due to the hydrophilicity of the lipid heads, water molecules can form an ordered hydration network near the membrane surfaces. Although our intra-axonal $R_{2}$ values as a function of theta have an extremum close to the magic angle, we observed a *maximum* in fibres with $\theta ∼54.7^{\circ },$ which contradicts the assumption of a *minimum* $R_{2}$ at the magic angle due to the minimal dephasing a dipole experiences from its neighbour because its field passes through zero (Chappell et al., 2004). Such apparent opposite behaviour has also been reported for the apparent water content (Schyboll et al., 2018) and myelin water fraction (Birkl et al., 2020). One potential cause may be that the MR-visible fraction of water increases at the magic angle, i.e. myelin water becoming visible at the TE used would necessarily increase the apparent $R_{2}$ estimated in other compartments as myelin water is not separately modelled. Notwithstanding the insignificant support of the isotropic model with $\Delta_{\text{AIC}}=76$ in the SFP analysis across the WM (Fig. 4), in the per-tract analysis the isotropic model was preferred (Fig. 6b).

The extra-axonal ${\hat{R}}_{2,\text{e}}\left(\theta \right)$ showed a $\sin^{4}\theta$-dependence on fibre orientation to ${\vec{B}}_{0}$. This is in agreement with predictions from a hollow cylindrical perturber in the quadratic dephasing regime (Knight, Dillon, Jarutyte, Kauppinen, 2017, Wharton, Bowtell, 2013, Yablonskiy, Haacke, 1994). The estimation of intra- and extra-axonal $T_{2}$ was performed on data with deliberately applied diffusion encoding gradients, which could explain the support for an additional $\sin^{2}\theta$ dependence accounting for the interaction with susceptibility differences (Knight et al., 2017) $\Delta_{\text{AIC}}=2$.

### 4.3 Signal-to-noise ratio

Our data showed similar differences for all subjects in the signal distribution between the two coil orientations in WM, but negligible differences in tSNR. The number of voxels with higher signal intensities was similar for both coil-orientations, however, the distribution of lower signal intensities was broader and flatter in WM for the tilted head orientation compared to the default head orientation. This was observed for all subjects as well as for the test-retest data from Participant 1. The intensity differences were relatively subtle and could be due to an additional small repositioning of the head relative to the coil in the tilted position, or being further away from isocentre, which in the case of gradient nonlinearties geometrically deforms voxels and potentially ‘smears out’ the signal. Nevertheless, these factors did not seem to affect the tSNR.

### 4.4 Test-retest reliability of results

The good agreement between test-retest signal distributions in Fig. 3 and the high correlation of fibre orientation to ${\vec{B}}_{0}$ and $R_{2}$-estimates in Fig. 8 demonstrates robustness of the setup. The tiltable coil has allowed us to achieve a better control over the participant’s head orientation during the experiment, to increase participant comfort, and to shorten the time for setup. Variability in the retest (especially in the tilted position) can be caused by additional unintended head rotation w.r.t. the coil, amongst others. Head rotation can be described by yaw (rotation around superior–inferior axis), pitch (rotation around left-right axis) and roll (rotation around anterior-posterior axis). The tiltable coil controls for the pitch re-orientation (Fig. 1A), therefore we expect that the major differences between test-retest data arise from differences in yaw and roll orientations. For the default coil-orientation only roll motion can change the fibre orientation to ${\vec{B}}_{0},$ since SI axis aligns with the ${\vec{B}}_{0}$-axis. In the tilted coil-orientation, yaw motion can additionally influence the fibre orientation θ to ${\vec{B}}_{0}$ and may explain the lower correlation between the test and the retest for both θ and $R_{2}$. Image registration could be considered to assess the $R_{2}$-variations between test-retest in greater detail. However, preliminary analyses revealed non-rigid residual deformations and challenges in registration between the non-tilted and tilted orientation (Tax, 2020), potentially caused by the relatively low image resolution and insufficient correction of geometrical distortions during preprocessing (e.g. due to interactions between susceptibility fields, eddy currents, and gradient nonlinearities). Hence, the registration would benefit from an additional high-resolution scan (here we opted for high SNR) and further developments in the pre-processing pipeline beyond this study.

### 4.5 Limitations and future work

Because we re-purposed an adjustable RF coil that was primarily designed for maximising patient comfort in clinical situations, the range of available coil orientations was necessarily limited. Nevertheless, the initial experiments presented here demonstrate the utility of this hardware design for uncovering compartmental orientation-effects *in vivo* and can motivate further hardware innovation in this domain. Here, as a proof-of-principle, we have focused on global and tract-wise characterisation across subjects leveraging the anatomical variation in pathway trajectories, because only two measurement points would be available for subject-wise characterisation on the voxel-level and any fit would be heavily influenced by noise. A larger range and number of coil orientations would allow the $\theta \;-\;R_{2}$ relationship to be elucidated more extensively, potentially on the voxel-level. Future work will explore the inclusion of additional head-orientations by asking the participant to further re-orient the head, including combinations of pitch and yaw which are more easily realised with the tiltable coil in the tilted coil-orientation.

A reasonable question is whether orientational dependence of $R_{2}$ could be studied *without* re-orienting the head but, instead, by exclusively relying on the natural twists and turns of anatomical structures within the brain and their relative orientation with respect to $B_{0}$. The potential challenge with this approach is that other anatomical factors may influence $R_{2}$. For example, we know that the mean axon diameter is larger in the corticospinal tract (CST), which runs predominantly in a superior-inferior orientation (parallel to the $B_{0}$ field when lying in the prone position), than in association pathways (which have a substantial component running along the anterior-posterior axis of the brain, which would be orthogonal to $B_{0}$). This anatomical variance in axon diameter is possibly driven by the distance over and/or the speed at which the axons need to carry action potentials. Thus, we can have correlation between orientation and axon diameter. Further, we know that surface-relaxation can influence $R_{2}$ and, notably, a slower $R_{2}$ is seen in the CST, which has already been hypothesised to be attributed to a reduced surface-relaxation effect (McKinnon and Jensen, 2019). Thus, relying on ‘anatomical variance’ alone may not unambiguously disentangle ‘true’ orientational effects from, e.g. differences in tissue microstructure (Kaden and Alexander, 2013). However, by reorienting exactly the *same* microstructure with respect to the magnetic field allows us to address this confound and explore ‘pure’ orientational differences in signal evolution. Moreover, in future work, and with more advanced coil designs that permit higher degrees of rotational freedom, local estimation of $R_{2}$ anisotropy may be possible and provides a new measure of tissue health. This is not possible without re-orienting the head.

The segment-wise analysis was adopted to study the overall effect of tilt and pools estimates across multiple voxels with the potential benefit of reducing the effects of noise through averaging, as is typically done in dMRI analyses. However, in such analyses it is generally not guaranteed that the number of voxels within an ROI is equal longitudinally in a single subject or across subjects. In our study this effect may be amplified because of the focus on single-fibre-population voxels, and we weighted segments according to their number of voxels in Fig. 7 while excluding segments with less than 3 voxels. We furthermore constrained the analysis to the ‘core’ segments of tracts (Yeatman et al., 2012) and confirmed that ∼95% of segments had an angular difference between the tilted and non-tilted position of within $20^{\circ },$ in agreement with the range of the tiltable coil. Comparison could be done voxel-wise but may be challenged by the aforementioned residual misalignment, which does not affect the segment-wise analysis done in native space. Moreover, even if both configurations could be aligned more optimally, it is not guaranteed that voxels flagged as single-fibre-populations overlap because of differences in partial volume effects. Overall, future analyses would benefit from including voxels beyond single-fibre-population voxels, i.e. $T_{2}$ characterisation per fibre population in crossing fibre voxels (Reymbaut et al., 2020, for example).

**Fig. 9:**
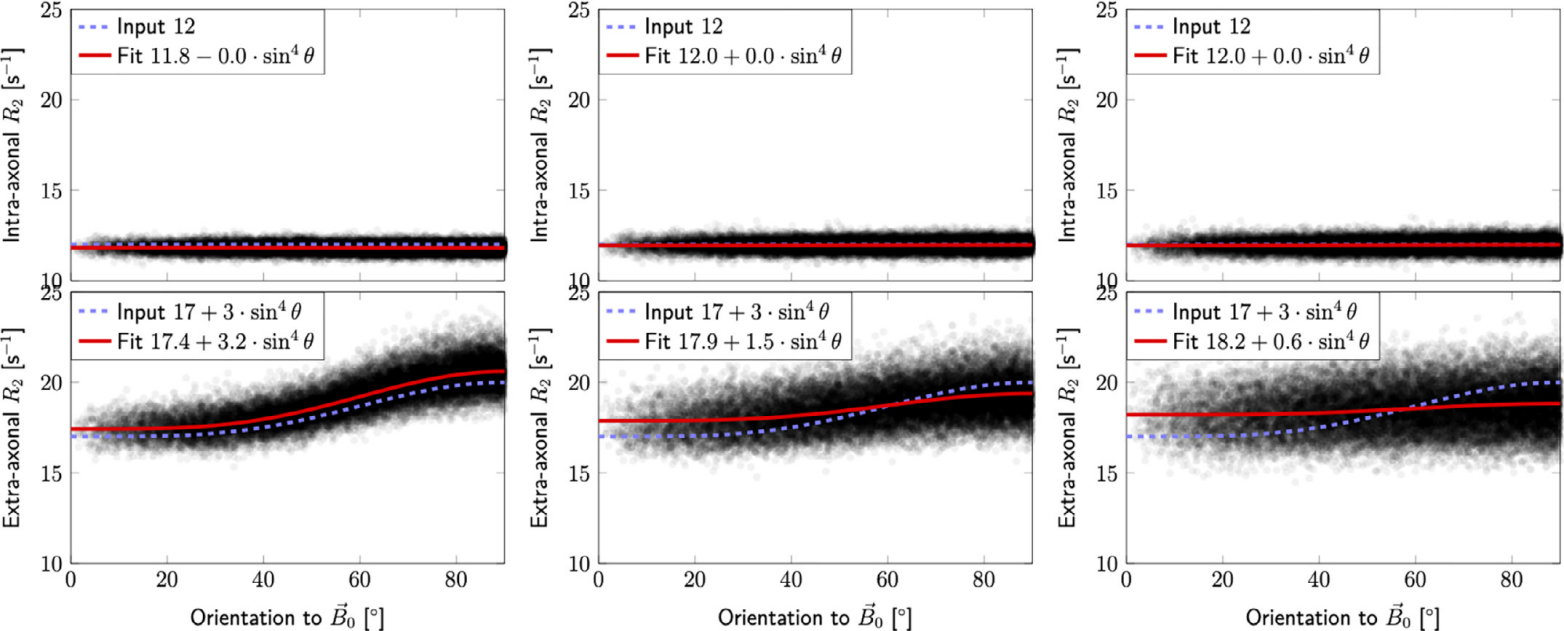
Simulated orientation dispersion of 0 (left), 0.16 (middle), and 0.32 (right) respectively, as described in Appendix A.2. Note that the estimation procedure (Appendix A.1) only captures the effect of orientation dispersion in the diffusion dimension. An under-estimation of $R_{2,\text{aniso}}$ can be observed as OD increases. Remaining simulation parameters (see also Supplementary materials): intra-axonal $R_{2,\text{i}}\left(\theta \right)=12s^{-1},$ anisotropic extra-axonal $R_{2,\text{e}}\left(\theta \right)=17+3\cdot \sin^{2}\theta ,$ signal fraction f=0.5, intra-axonal parallel, and extra-axonal parallel and perpendicular diffusivities $D_{∥,\text{i}}=2.5\mu {\text{m}}^{2}/\text{ms},$$D_{∥,\text{e}}=2\mu {\text{m}}^{2}/\text{ms},$ and $D_{⊥,\text{e}}=0.8\mu {\text{m}}^{2}/\text{ms},$ respectively. Gaussian noise was added to the signal with the default SNR input parameters of SNR=100 on the S(0,0) signal corresponding to SNR≈50 on the S(0,54) signal assuming $T_{2}\approx 70$ ms. The angles and number of points were taken from the data analysed in this work (cf Fig. 4). An under-estimation of $R_{2,\text{aniso}}$ can be observed as OD increases.

Fibre dispersion can be a confounding factor, and the majority of works investigating $T_{2}$ orientation dependence are based on DT-MRI and do not include a specific measure for fibre crossing and dispersion. We have minimised the effect of crossing fibres by filtering voxels based on their fODF. We further investigate the effect of fibre orientation dispersion by considering a distribution of orientation-dispersed compartments according to a Watson distribution, where each sub-compartment (e.g. each extra-axonal zeppelin) can separately exhibit $R_{2}$-orientation dependence (Appendix A.2). Fig. 9 confirms the expectation that if orientation dispersion increases, $R_{2,\text{aniso}}$ is under-estimated. We have further investigated this effect in our data by excluding voxels with an ‘orientation coherence’ parameter $p_{2}$ below 0.5 (Appendix A.1.1), revealing that the extra-axonal ${\hat{R}}_{2,\text{aniso}}$ is increased (Supplementary Figure S4). We aim to investigate this further in future work with more head orientations. Similarly, in the segment-wise analysis, orientational dispersion between voxels in each tract segment could influence the averaged angle- and relaxation rate-estimates. In Supplementary Figs. S7 and S8 we have explored the effect of the voxel-wise threshold on $p_{2}$ and the segment-wise angular threshold; imposing stricter thresholds did not alter the overall result and still revealed a significant effect of tilt in the mono-exponential and extra-axonal $R_{2},$ while favouring the isotropic model for intra-axonal $R_{2}$.

The use of strong gradients can lead to a deviation from the Gaussian behaviour of compartments in diffusion MRI (Grebenkov, 2018). In this work we sought primarily to disentangle compartmental $T_{2}$ based on differences in apparent diffusion anisotropy between the compartments, and hence the focus was less on characterisation of the diffusion parameters. In Supplementary Figure S13 we have investigated the effect of introducing a finite cylinder radius, thereby breaking the stick assumption (Veraart et al., 2020). We found only minimal effect on the estimation of $R_{2,\text{iso}}$ and $R_{2,\text{aniso}}$. Nevertheless, deviations from Gaussian behaviour at strong gradients open up new opportunities to probe microstructure, and the characterisation of this regime is an important avenue moving forward as access to such MRI systems becomes more readily available.

The estimation in this work was performed with least squares approaches on magnitude images, which can yield biased results if the underlying noise-distribution is non-Gaussian. We have aimed to ameliorate this issue by adopting a signal transformation framework (Koay et al., 2009a) which relies on an estimate of the signal and the noise standard deviation. While previously tested in simulations (Tax et al., 2020), we acknowledge that errors in signal- and noise estimates can propagate (we particularly observed this with apparent over-estimation of noise estimates using different procedures) and parallel imaging can result in spatially varying noise across the slice. In future work, this issue is more ideally addressed by using complex data (Eichner, Cauley, Cohen-Adad, Möller, Turner, Setsompop, Wald, 2015, Pizzolato, Gilbert, Thiran, Descoteaux, Deriche, 2020) or Maximum Likelihood estimation. Omitting this step in the preprocessing pipeline did not seem to affect the overall observations of orientation-dependence (Figs. 4 and 7).

The scan time of the protocol in the current study was approximately one hour per coil-orientation. While this is acceptable for, e.g., methodological research studies on a few participants, such scan times become prohibitive in larger cohorts and populations that have difficulties remaining still in the scanner. Advances in experiment-design optimisation (Alexander, 2008, Hutter, Slator, Christiaens, Teixeira, Roberts, Jackson, Price, Malik, Hajnal, 2018, Lampinen, Szczepankiewicz, Mårtensson, van Westen, Hansson, Westin, Nilsson, 2020; Tax et al., 2021) can drastically shorten acquisition time while keeping the most crucial information, even in multi-dimensional correlation MRI experiments.

The individual images were corrected for participant motion, which could also involve head re-orientation with respect to the magnetic field during the course of the scan. Participants in this study were experienced MR experimental volunteers, therefore the maximum involuntary rotation beyond that imposed by the tilting of the coil rarely exceeded $1.5^{\circ }$. However, when considering the application of this method on less compliant subjects, subject motion could become a confounding factor.

Finally, the nature of the EPI readout (the most common readout in dMRI) potentially leads to partially incomplete refocusing and thus residual $T_{2}^{*}$-effects in the data. Here, these effects were considered minimal due to a reduced EPI duration because of the relatively low resolution and parallel imaging. Yet, these effects can be reduced even further by implementing non-Cartesian readout techniques in future.

The observed orientation-dependence can have important ramifications for future analyses and methods development. For example at the tract-level $T_{2}$ relaxation is usually treated as a scalar and often assumed to be invariant along the length of a tract. However, even if the intrinsic microstructural properties are invariant along the tract, our results show that the apparent $T_{2}$ will not and future work could extend ‘global’ tractography frameworks (Barakovic et al., 2021, Daducci, Dal Palù, Lemkaddem, Thiran, 2015) that attempt to estimate a microstructural parameter *per* streamline to account for this. This work has focused on the orientation dependence of $T_{2},$ but previous work has shown that local susceptibility and transverse relaxation rate also affects the apparent diffusion coefficient, i.e. it decreases with time (Novikov et al., 2018). Future work will explore the orientation dependence of diffusion measures and its effect on dMRI analyses.

## 5 Conclusion

Using a novel combination of a tiltable RF-coil and ultra-strong magnetic field gradients in combined diffusion–relaxation experiments, we have demonstrated a separation of compartmental (intra- and extra-axonal) $T_{2}$-orientational dependencies, based on their diffusion anisotropy. The enhanced tissue compartmental specificity afforded by the current framework should assist in the formulation of more complete models of white matter microstructure, and improved understanding of disease, whether through enhanced ability to *interpret* signal changes in multi-dimensional experiments, or indeed through improved sensitivity to tissue damage/change by isolating microstructural changes to one particular sub-compartment of tissue. Our findings furthermore have consequences for longitudinal- or group-studies of apparent $T_{2}$ and diffusion MRI features, as the positioning in the scanner introduces additional variability. This work motivates the further development of hardware - i.e., a coil with more degrees of freedom (including more axes of rotation) and a wider range of rotations about any those axes.

## CRediT authorship contribution statement

**Chantal M.W. Tax:** Conceptualization, Methodology, Software, Formal analysis, Investigation, Writing - original draft, Visualization, Supervision, Funding acquisition. **Elena Kleban:** Conceptualization, Methodology, Software, Formal analysis, Investigation, Writing - original draft, Visualization, Supervision. **Maxime Chamberland:** Methodology, Software, Formal analysis, Writing - review & editing, Visualization. **Muhamed Baraković:** Conceptualization, Methodology, Writing - review & editing. **Umesh Rudrapatna:** Conceptualization, Methodology, Writing - review & editing. **Derek K. Jones:** Conceptualization, Methodology, Resources, Writing - review & editing, Supervision, Funding acquisition.

## Appendix A Compartmental model

### A1 No $R_{2}$-orientation dependence

The signal in the long-time limit and in the case of no $R_{2}$-orientation dependence can be described as

(A.1) $$\begin{array}{ccc} & & S\left(\text{TE},b,b_{\Delta },\theta_{g},\phi_{g}\right)=S_{0}\cdot [\int_{0}^{\infty }\int_{0}^{\infty }\int_{0}^{\infty }\int_{0}^{\pi }\int_{0}^{2\pi }\\ & & \;\times k\left(\text{TE},b,b_{\Delta },\theta_{g},\phi_{g},\Theta ,\Phi ,D_{∥},D_{⊥},R_{2}\right)\\ & & \;\times \;p\left(\Theta ,\Phi ,D_{∥},D_{⊥},R_{2}\right)d\Phi \sin\Theta d\Theta dD_{∥}dD_{⊥}dR_{2}]\; \end{array}$$

with p the continuous joint probability distribution of $R_{2}$ and axially symmetric diffusion tensors (parameterised by parallel diffusivity $D_{∥},$ perpendicular diffusivity $D_{⊥}$ and first eigenvector direction (Θ,Φ)), and the kernel k mapping the distribution onto the detected signal (de Almeida Martins et al., 2020b). The acquisition is characterised by echo time TE and the axially symmetric positive semi-definite b-matrix B with b-value $b=\text{Tr}\left(B\right)=b_{∥}+2b_{⊥}$ and $b_{\Delta }=\left(b_{∥}-b_{⊥}\right)/b,$ where $b_{∥}$ and $b_{⊥}$ are the eigenvalues corresponding to the eigenvectors along and perpendicular to the symmetry axis $(\theta_{g},\phi_{g}),$ respectively (Eriksson, Lasic, Topgaard, 2013, Topgaard, 2017, Westin, Knutsson, Pasternak, Szczepankiewicz, Özarslan, van Westen, Mattisson, Bogren, O’Donnell, Kubicki, Topgaard, Nilsson, 2016).

Lampinen et al. (2020); McKinnon and Jensen (2019); Veraart et al. (2018) adopt the following form for the joint probability distribution (Tax, 2020)

(A.2) $$\begin{array}{ccc} & & p\left(\Theta ,\Phi ,D_{∥},D_{⊥},R_{2}\right)=p\left(\Theta ,\Phi \right)\left(f\delta (R_{2}-R_{2,\text{i}},D_{⊥},D_{∥}-D_{∥,\text{i}}\right)\\ & & +\left(1-f\right)\delta \left(R_{2}-R_{2,\text{e}},D_{⊥}-D_{⊥,\text{e}},D_{∥}-D_{∥,\text{e}}\right))\; \end{array}$$

where the delta distribution is defined as $\int_{-\infty }^{\infty }\delta \left(x-a\right)f\left(x\right)dx=f\left(a\right)$ and we abuse the same notation for multivariate distributions. This reflects a two-compartment model with the intra-axonal compartment parameterised by $R_{2,\text{i}}$ and $D_{∥,\text{i}},$ and the extra-axonal compartment parameterised by $R_{2,\text{e}},D_{⊥,\text{e}},$ and $D_{∥,\text{e}}$. f is the signal fraction of the intra-axonal compartment and p(Θ,Φ) the fibre orientation distribution function. With the kernel defined as

(A.3) $$\begin{array}{ccc} & & k\left(\text{TE},b,b_{\Delta },\theta_{g},\phi_{g},\Theta ,\Phi ,D_{∥},D_{⊥},R_{2}\right)=\exp\left(-\text{Tr}(\mathrm{BD})\right)\exp\left(-\text{TE}R_{2}\right)\\ & & =\exp(\frac{1}{3}bb_{\Delta }\left(D_{∥}-D_{⊥}\right)-\frac{1}{3}b\left(D_{⊥}+2D_{∥}\right)\\ & & \;-bb_{\Delta }{\left(\left(\theta_{g},\phi_{g}\right)\cdot \left(\Theta ,\Phi \right)\right)}^{2}\left(D_{∥}-D_{⊥}\right))\exp\left(-\text{TE}R_{2}\right)\; \end{array}$$

this reduces down to

(A.4) $$\begin{array}{ccc} & & S(\text{TE},b,b_{\Delta },\theta_{g},\phi_{g})\;\\ & & =S_{0}\cdot [f\int_{0}^{\pi }\int_{0}^{2\pi }\exp\left(-bb_{\Delta }{\left(\left(\theta_{g},\phi_{g}\right)\cdot \left(\Theta ,\Phi \right)\right)}^{2}D_{∥,\text{i}}\right)p\left(\Theta ,\Phi \right)d\Phi \sin\Theta d\Theta \\ & & \;\times \exp\left(\frac{1}{3}bb_{\Delta }D_{∥,\text{i}}-\frac{2}{3}bD_{∥,\text{i}}\right)\exp\left(-\text{TE}R_{2,\text{i}}\right)\\ & & \;+\left(1-f\right)\int_{0}^{\pi }\int_{0}^{2\pi }\exp\left(-bb_{\Delta }{\left(\left(\theta_{g},\phi_{g}\right)\cdot \left(\Theta ,\Phi \right)\right)}^{2}\left(D_{∥,\text{e}}-D_{⊥,\text{e}}\right)\right)\\ & & \;\times p\left(\Theta ,\Phi \right)d\Phi \sin\Theta d\Theta \exp\left(\frac{1}{3}bb_{\Delta }\left(D_{∥,\text{e}}-D_{⊥,\text{e}}\right)-\frac{1}{3}b\left(D_{⊥,\text{e}}+2D_{∥,\text{e}}\right)\right)\\ & & \;\times \exp\left(-\text{TE}R_{2,\text{e}}\right)]. \end{array}$$

This signal equation was used to generate signals for simulations involving orientation dispersion, where p(Θ,Φ) was parameterised by a Watson distribution:

(A.5) $$p_{\text{Watson}}\left(\Theta ,\Phi \right)=c\left(\kappa \right)\exp{\left(\kappa \left(\theta_{\mu },\phi_{\mu }\right)\cdot \left(\Theta ,\Phi \right)\right)}^{2},$$

with $\left(\theta_{\mu },\phi_{\mu }\right)$ the symmetry axis of the Watson distribution, c(κ) a normalisation constant, and orientation dispersion $\text{OD}=\frac{2}{\pi }\arctan\left(\frac{1}{\kappa }\right)$.

#### A1.1 Implementation and estimation

Veraart et al. (2018), Lampinen et al. (2020) project the signal onto real spherical harmonics, with the difference being that Veraart et al. (2018) factors out p(Θ,Φ) by the computation of rotational invariants per b- and TE shell. Briefly, spherical convolution with an axially symmetric kernel k can be written as (Kennedy et al., 2011):

(A.6) $$S\left(\theta ,\phi \right)=\sum_{l}\sum_{m=-l}^{l}\sqrt{\frac{4\pi }{2l+1}}k_{l0}p_{lm}Y_{lm}\left(\theta ,\phi \right),$$

with $k_{lm}=0$ if m≠0 and

(A.7) $$k_{l0}=\sqrt{\pi (2l+1)}\int_{0}^{\pi }k\left(\theta \right)P_{l}^{0}\left(\cos\theta \right)\sin\theta d\theta ,$$

(A.8) $$Y_{l}^{m}\left(\theta ,\phi \right)=\sqrt{\frac{2l+1}{4\pi }\frac{(l-m)!}{(l+m)!}}P_{l}^{m}\left(\cos\theta \right)e^{im\phi }$$

complex spherical harmonics,

(A.9) $$\begin{array}{c} Y_{lm}\left(\theta ,\phi \right)=\left\{ \begin{array}{cc} \sqrt{2}\text{Im}\left[Y_{l}^{-m}\right], & \text{if}m<0\\ Y_{l}^{0}, & \text{if}m=0\\ \sqrt{2}\text{Re}\left[Y_{l}^{m}\right], & \text{if}m>0 \end{array}\right. \end{array}$$

real spherical harmonics, and $P_{l}^{m}$ associated Legendre polynomials. Substituting the definition for k in Eq. A.3 gives

(A.10) $$\begin{array}{cc} k_{l0} & =\sqrt{\pi (2l+1)}\exp\left(-\text{TE}R_{2}\right)\exp\left(\frac{1}{3}bb_{\Delta }\left(D_{∥}-D_{⊥}\right)-\frac{1}{3}b\left(D_{⊥}+2D_{∥}\right)\right)\\ & \;\times \int_{0}^{\pi }\exp\left(-bb_{\Delta }\cos^{2}\theta \left(D_{∥}-D_{⊥}\right)\right)P_{l}^{0}\left(\cos\theta \right)\sin\theta d\theta \\ & =\sqrt{\pi (2l+1)}\exp\left(-\text{TE}R_{2}\right)\exp\left(\frac{1}{3}bb_{\Delta }\left(D_{∥}-D_{⊥}\right)-\frac{1}{3}b\left(D_{⊥}+2D_{∥}\right)\right)\\ & \;\times 2\int_{0}^{1}\exp\left(-bb_{\Delta }x^{2}\left(D_{∥}-D_{⊥}\right)\right)P_{l}^{0}\left(x\right)dx\; \end{array}$$

where the latter is obtained by substituting x=cosθ and dx=−sinθdθ. The forward equation for the signal then becomes

(A.11) $$\begin{array}{cc} S & (\text{TE},b,b_{\Delta },\theta_{g},\phi_{g})\;\\ & =S_{0}\cdot [f\exp\left(\frac{1}{3}bb_{\Delta }D_{∥,\text{i}}-\frac{2}{3}bD_{∥,\text{i}}\right)\exp\left(-\text{TE}R_{2,\text{i}}\right)\\ & \;\;\times \sum_{l}\sum_{m=-l}^{l}\sqrt{\frac{4\pi }{2l+1}}\sqrt{\pi (2l+1)}2\int_{0}^{1}\exp\left(-bb_{\Delta }x^{2}D_{∥,\text{i}}\right)P_{l}^{0}\left(x\right)dx\;p_{lm}Y_{lm}\left(\theta_{g},\phi_{g}\right)\\ & \;\;+\left(1-f\right)\exp\left(\frac{1}{3}bb_{\Delta }\left(D_{∥,\text{e}}-D_{⊥,\text{e}}\right)-\frac{1}{3}b\left(D_{⊥,\text{e}}+2D_{∥,\text{e}}\right)\right)\exp\left(-\text{TE}R_{2,\text{e}}\right)\\ & \;\;\times \sum_{l}\sum_{m=-l}^{l}\sqrt{\frac{4\pi }{2l+1}}\sqrt{\pi (2l+1)}2\int_{0}^{1}\exp\left(-bb_{\Delta }x^{2}\left(D_{∥,\text{e}}-D_{⊥,\text{e}}\right)\right)\\ & \;\;\times P_{l}^{0}\left(x\right)dx\;p_{lm}Y_{lm}\left(\theta_{g},\phi_{g}\right)]\\ & =4\pi S_{0}\cdot [f\exp\left(\frac{1}{3}bb_{\Delta }D_{∥,\text{i}}-\frac{2}{3}bD_{∥,\text{i}}\right)\exp\left(-\text{TE}R_{2,\text{i}}\right)\\ & \;\;\times \sum_{l}\sum_{m=-l}^{l}\int_{0}^{1}\exp\left(-bb_{\Delta }x^{2}D_{∥,\text{i}}\right)P_{l}^{0}\left(x\right)dx\;p_{lm}Y_{lm}\left(\theta_{g},\phi_{g}\right)\\ & \;\;+\left(1-f\right)\exp\left(\frac{1}{3}bb_{\Delta }\left(D_{∥,\text{e}}-D_{⊥,\text{e}}\right)-\frac{1}{3}b\left(D_{⊥,\text{e}}+2D_{∥,\text{e}}\right)\right)\exp\left(-\text{TE}R_{2,\text{e}}\right)\\ & \;\;\times \sum_{l}\sum_{m=-l}^{l}\int_{0}^{1}\exp\left(-bb_{\Delta }x^{2}\left(D_{∥,\text{e}}-D_{⊥,\text{e}}\right)\right)P_{l}^{0}\left(x\right)dx\;p_{lm}Y_{lm}\left(\theta_{g},\phi_{g}\right)]. \end{array}$$

In the current work we acquired measurements with $b_{\Delta }=1$ and use $l_{\max}=2$ as in Lampinen et al. (2020), and we estimate $S_{0},f,D_{∥,\text{i}},D_{∥,\text{e}},D_{⊥,\text{e}},R_{2,\text{i}},R_{2,\text{e}},p_{2-2},p_{2-1},p_{20},p_{21},$ and $p_{22},$ while we set $p_{00}=Y_{00}=\frac{1}{\sqrt{4\pi }}$. A measure of ‘orientation coherence’ can be computed as $p_{2}=\sqrt{\frac{4\pi }{5}}\sqrt{\sum_{m}{\left|p_{2m}\right|}^{2}}$ (Novikov, Veraart, Jelescu, Fieremans, 2018, Reisert, Kellner, Dhital, Hennig, Kiselev, 2017).

### A2 $R_{2}$-orientation dependence

If each compartment separately can exhibit orientation dependence according to the most general form $R_{2}\left(\theta \right)=R_{2,\text{iso}}+R_{2,{\text{aniso}}_{1}}\cdot \sin^{2}\theta +R_{2,{\text{aniso}}_{2}}\cdot \sin^{4}\theta$ where in this section $\sin\theta =∥\left(\theta_{B_{0}},\phi_{B_{0}}\right)\times \left(\Theta ,\Phi \right)∥$ and $\left(\theta_{B_{0}},\phi_{B_{0}}\right)$ is the direction of $B_{0},$ then the signal and kernel become

(A.12) $$\begin{array}{cc} & S\left(\text{TE},b,b_{\Delta },\theta_{g},\phi_{g},\theta_{B_{0}},\phi_{B_{0}}\right)\;=S_{0}\cdot [\int_{0}^{\infty }\int_{0}^{\infty }\int_{0}^{\infty }\int_{0}^{\infty }\int_{0}^{\infty }\int_{0}^{\pi }\int_{0}^{2\pi }\\ & \;\times \;k\left(\text{TE},b,b_{\Delta },\theta_{g},\phi_{g},\theta_{B_{0}},\phi_{B_{0}},\Theta ,\Phi ,D_{∥},D_{⊥},R_{2,\text{iso}},R_{2,{\text{aniso}}_{1}},R_{2,{\text{aniso}}_{2}}\right)\\ & \;\times \;p\left(\Theta ,\Phi ,D_{∥},D_{⊥},R_{2,\text{iso}},R_{2,{\text{aniso}}_{1}},R_{2,{\text{aniso}}_{2}}\right)\\ & \;\times \;d\Phi \sin\Theta d\Theta dD_{∥}dD_{⊥}dR_{2,\text{iso}}dR_{2,{\text{aniso}}_{1}}dR_{2,{\text{aniso}}_{2}}]\; \end{array}$$

(A.13) $$\begin{array}{cc} k & \left(\text{TE},b,b_{\Delta },\theta_{g},\phi_{g},\theta_{B_{0}},\phi_{B_{0}},\Theta ,\Phi ,D_{∥},D_{⊥},R_{2,\text{iso}},R_{2,{\text{aniso}}_{1}},R_{2,{\text{aniso}}_{2}}\right)\\ & =\exp\left(-\text{Tr}(\mathrm{BD})\right)\exp\left(-\text{TE}\left(R_{2,\text{iso}}+R_{2,{\text{aniso}}_{1}}\cdot \sin^{2}\theta +R_{2,{\text{aniso}}_{2}}\cdot \sin^{4}\theta \right)\right)\\ & =\exp\left(\frac{1}{3}bb_{\Delta }\left(D_{∥}-D_{⊥}\right)-\frac{1}{3}b\left(D_{⊥}+2D_{∥}\right)-bb_{\Delta }{\left(\left(\theta_{g},\phi_{g}\right)\cdot \left(\Theta ,\Phi \right)\right)}^{2}\left(D_{∥}-D_{⊥}\right)\right)\\ & \;\times \exp\left(-\text{TE}\left(R_{2,\text{iso}}+R_{2,{\text{aniso}}_{1}}\cdot \sin^{2}\theta +R_{2,{\text{aniso}}_{2}}\cdot \sin^{4}\theta \right)\right). \end{array}$$

Following the hollow-cylinder model only the extra-axonal compartment exhibits $\sin^{4}\theta$-dependence, giving

(A.14) $$\begin{array}{cc} p & \left(\Theta ,\Phi ,D_{∥},D_{⊥},R_{2,\text{iso}},R_{2,{\text{aniso}}_{1}},R_{2,{\text{aniso}}_{2}}\right)\\ & =p\left(\Theta ,\Phi \right)[f\delta \left(R_{2}-R_{2,\text{iso,i}},R_{2,{\text{aniso}}_{1}},R_{2,{\text{aniso}}_{2}},D_{⊥},D_{∥}-D_{∥,i}\right)\\ & \;+\left(1-f\right)\delta (R_{2}-R_{2,\text{iso,e}},R_{2,{\text{aniso}}_{1}},R_{2,{\text{aniso}}_{2}}\\ & \;-R_{2,\text{aniso,e}},D_{⊥}-D_{⊥,e},D_{∥}-D_{∥,e})]. \end{array}$$

Plugging these equations gives

(A.15) $$\begin{array}{cc} S & (\text{TE},b,b_{\Delta },\theta_{g},\phi_{g},\theta_{B_{0}},\phi_{B_{0}})\;\\ & =S_{0}\cdot [\int_{0}^{\pi }\int_{0}^{2\pi }\exp\left(-bb_{\Delta }{\left(\left(\theta_{g},\phi_{g}\right)\cdot \left(\Theta ,\Phi \right)\right)}^{2}D_{∥,\text{i}}\right)d\Phi \sin\Theta d\Theta \\ & \;\;\times \exp\left(\frac{1}{3}bb_{\Delta }D_{∥,\text{i}}-\frac{2}{3}bD_{∥,\text{i}}\right)\exp\left(-\text{TE}R_{2,\text{iso,i}}\right)\\ & \;\;+\int_{0}^{\pi }\int_{0}^{2\pi }\exp\left(-bb_{\Delta }{\left(\left(\theta_{g},\phi_{g}\right)\cdot \left(\Theta ,\Phi \right)\right)}^{2}\left(D_{∥,\text{e}}-D_{⊥,\text{e}}\right)\right)\\ & \;\;\times \exp\left(-\text{TE}\left(R_{2,\text{aniso},\text{e}}{∥\left(\theta_{B_{0}},\phi_{B_{0}}\right)\times \left(\Theta ,\Phi \right)∥}^{4}\right)\right)p\left(\Theta ,\Phi \right)d\Phi \sin\Theta d\Theta \\ & \;\;\times \exp\left(\frac{1}{3}bb_{\Delta }\left(D_{∥,\text{e}}-D_{⊥,\text{e}}\right)-\frac{1}{3}b\left(D_{⊥,\text{e}}+2D_{∥,\text{e}}\right)\right)\exp\left(R_{2,\text{iso},\text{e}}\right)]. \end{array}$$

## Appendix B Trigonometric relations

We here derive some equivalent trigonometric relations used in the literature and in Table 1.

(B.1) $$\cos(2\theta )=\cos^{2}\theta -\sin^{2}\theta =1-2\sin^{2}\theta$$

(B.2) $$\cos(4\theta )=2\cos^{2}\left(2\theta \right)-1\overset{\text{Equation}\;\text{B.1}}{=}8\sin^{4}\theta -8\sin^{2}\theta +1$$

1. A $\sin^{2}\theta$ relationship Bender and Klose (2010) can equivalently be written in terms of cos(2θ):
  $$\begin{array}{cc} a+b\cdot \cos(2\theta ) & =a+b-2b\cdot \sin^{2}\theta \\ & =A+B\cdot \sin^{2}\theta , \end{array}$$
  where A=a+b and B=−2b are independent parameters.
2. Detailed studies at high field have shown an additional cos4θ dependence (Denk, Torres, MacKay, Rauscher, 2011, Lee, van Gelderen, Kuo, Merkle, Silva, Duyn, 2011). Using Eq. B.1 and Eq. B.2:
  $$\begin{array}{cc} & a+b\cos(2\theta )+c\cos(4\theta )\\ & \;=a+b\cdot \left(1-2\sin^{2}\theta \right)+c\cdot \left(8\sin^{4}\theta -8\sin^{2}\theta +1\right)\\ & \;=a+b-2b\sin^{2}\theta +8c\cdot \sin^{4}\theta -8c\cdot \sin^{2}\theta +c\\ & \;=\left(a+b+c\right)+\left(-2b-8c\right)\cdot \sin^{2}\theta +8c\cdot \sin^{4}\theta \\ & \;=A+B\cdot \sin^{2}\theta +C\cdot \sin^{4}\theta , \end{array}$$
  where A=a+b+c, B=−2b−8c and C=8c are independent parameters.
3. The magic angle effect can be written as:
  $$\begin{array}{cc} a+b\cdot {\left(3\cos^{2}\theta -1\right)}^{2} & =a+b\cdot {\left(3\left(1-\sin^{2}\theta \right)-1\right)}^{2}\\ & =a+b\cdot {\left(2-3\sin^{2}\theta \right)}^{2}\\ & =a+b\cdot (4-6\sin^{2}\theta +9\sin^{4}\theta )\\ & =a+4b-6b\cdot \sin^{2}\theta +9b\cdot \sin^{4}\theta \\ & =A+B\cdot \sin^{2}\theta -1.5B\cdot \sin^{4}\theta , \end{array}$$
  where A=a+4b and B=−6b are independent parameters, while the coefficients for $\sin^{2}\theta$ and $\sin^{4}\theta$ depend on each other.
  The magic angle expression can be also re-written such that the second coefficient is multiplied with a θ-dependent expression which is 0 when θ=0:
  $$\begin{array}{cc} a+b\cdot {\left(3\cos^{2}\theta -1\right)}^{2} & =A-4b+b\cdot {\left(3\cos^{2}\theta -1\right)}^{2}\\ & =A-4b\cdot \left[1-\frac{1}{4}{\left(3\cos^{2}\theta -1\right)}^{2}\right] \end{array}$$
4. Finally, a $\sin^{4}\theta$-relationship can be rewritten as:
  $$\begin{array}{cc} a+b\sin^{4}\theta & =a+\frac{b}{8}\cdot \left(\cos\left(4\theta \right)+8\sin^{2}\theta -1\right)\\ & =a+b\cdot \left(\frac{1}{8}\cos\left(4\theta \right)+\sin^{2}\theta -\frac{1}{8}\right)\\ & =a+b\cdot \left(\frac{1}{8}\cos\left(4\theta \right)+\frac{1-\cos(2\theta )}{2}-\frac{1}{8}\right)\\ & =\left(a+\frac{3}{8}b\right)-\frac{1}{2}b\cdot \cos\left(2\theta \right)+\frac{1}{8}b\cdot \cos\left(4\theta \right)\\ & =A+B\cos\left(2\theta \right)-\frac{B}{4}\cos\left(4\theta \right) \end{array}$$

## Appendix C Supplementary materials

Supplementary Data S1 Supplementary Raw Research Data. This is open data under the CC BY license http://creativecommons.org/licenses/by/4.0/ Supplementary Data S1
