# Supplementary material for: Measuring compartmental T2-orientational dependence in human brain white matter using a tiltable RF coil and diffusion-T2 correlation MRI
Source: Neuroimage. 2021 Aug 1;236:117967. doi: 10.1016/j.neuroimage.2021.117967 (PMC8270891; doi:10.1016/j.neuroimage.2021.117967)
Supplement: Supplementary Data S1 — Supplementary Raw Research Data. This is open data under the CC BY license http://creativecommons.org/licenses/by/4.0/ [file mmc1.pdf]

Supplementary data for manuscript
"Measuring compartmental T_2-orientational dependence in human brain white matter using a tiltable RF coil and diffusion-T_2 correlation MRI"

Chantal M.W. Tax[a,b,1,*], Elena Kleban[c,1], Maxime Chamberland[c], Muhamed Baraković[c,d,e], Umesh Rudrapatna[c], Derek K. Jones[c,f]

[a] *Cardiff University Brain Research Imaging Centre (CUBRIC), School of Physics and Astronomy, Cardiff University, Queen's Building, The Parade, Cardiff, Wales, UK, CF24 3AA*
[b] *University Medical Center Utrecht, Utrecht University, Heidelberglaan 100, 3584 CX Utrecht, The Netherlands*
[c] *Cardiff University Brain Research Imaging Centre (CUBRIC), School of Psychology, Cardiff University, Maindy Road, Cardiff, CF24 4HQ, UK*
[d] *Signal Processing Laboratory 5, Ecole Polytechnique Federale de Lausanne, Lausanne, Switzerland*
[e] *Translational Imaging in Neurology Basel, Department of Biomedical Engineering, University Hospital Basel, Basel, Switzerland*
[f] *Mary MacKillop Institute for Health Research, Faculty of Health Sciences, Australian Catholic University, Melbourne, Victoria, 3065, Australia*

1. Literature overview

Different mechanisms have been suggested to explain T_2^* and T_2-orientation dependence, including susceptibility effects from myelin, de-oxyhemoglobin in vasculature, tissue iron and magic-angle effects (Cherubini et al., 2009; Denk et al., 2011). To study susceptibility effects in T_2^* GRE experiments, the system is commonly assumed to be in the static dephasing regime where spin diffusion can be ignored. In contrast, SE experiments refocus such static dephasing, and it is the dynamic interaction between diffusion and mesoscopic field inhomogeneities that is thought to affect the apparent T_2.

We will first summarise literature on susceptibility effects and iron in the static regime as origins of T_2^*-anisotropy, and then outline their reported effects on T_2-orientation dependence. Finally we will briefly discuss the magic angle effect which affects T_2 and thus also T_2^*.

1.1. T_2^* anisotropy and the static dephasing regime

Yablonskiy & Haacke (1994) derived theory for NMR signal behaviour for the static dephasing regime, which in the case of parallel cylinders (e.g. myelinated axons or blood vessels), with isotropic magnetic susceptibility, results in a $\sin^2\theta$-dependence of the characteristic frequency shift $\Delta\omega$, and thus a $\sin^2\theta$-dependence of R_2' for long TE (TE > $(\Delta\omega)^{-1}$, 'linear regime') and a $\sin^4\theta$-dependence for short TE

* Corresponding author
Email address: TaxC@cardiff.ac.uk (Chantal M.W. Tax)
[1] These authors contributed equally to this work

(TE < $(\Delta\omega)^{-1}$, 'quadratic regime'). Bender & Klose (2010) found a good fit of a $\sin^2\theta$-dependence to their R_2^*-values from *in vivo* human brain data at $3\,$T with a TE-range of $26\ldots86\,$ms.

Several studies proposed myelin susceptibility as a primary source of T_2^*-orientational dependence. Wharton & Bowtell (2013); Gil et al. (2016) argued that the signal arising from the extra-axonal compartment is in the short-time quadratic regime, and they therefore expect a pure $\sin^4\theta$-dependence. Gil et al. (2016) indeed found a better fit compared to $\sin^2\theta$ in *in vivo* human brain at $3\,$T with TE-range of $5\ldots77\,$ms. Other studies proposed a combined $A + B\cdot\sin^2\theta + C\cdot\sin^4\theta$- dependence, or $a + b\cdot\cos2\theta + c\cdot\cos4\theta$-dependence, which are equivalent (Appendix B). Lee et al. (2011); Oh et al. (2013) studied the orientational-dependence in WM in fixed human brain at $7\,$T (with TE above $4.6\,$ms and TE range of $4\ldots39\,\mathrm{m}s$ respectively), and suggest that the additional $\cos4\theta$-dependence can be explained by susceptibility anisotropy originating from myelin. Dibb & Liu (2017) use a $\sin^2\theta$-relationship of R_2^* in conjunction with anisotropic susceptibility mapping to map fibre orientation, but discuss that including a $\sin^4\theta$ term would technically be more correct. Anisotropic susceptibility from the myelin sheath was demonstrated by Lee et al. (2010) to dominate the micro-compartmentalisation effects in an experiment involving re-orientation of postmortem tissue from the human corpus callosum in the magnetic field. Li et al. (2012) performed susceptibility tensor imaging in the human brain *in vivo* concluding that cylindrically aligned lipid molecules in myelin are the main source of bulk susceptibility anisotropy. Wharton & Bowtell (2012) proposed a hollow cylinder fibre model which described the myelin sheath as an infinitely long hollow cylinder, and found that it was necessary to include anisotropic susceptibility in the model to explain the GRE signal evolution and the orientation-dependent frequency difference. Finally, van Gelderen et al. (2015) provided an independent characterisation of the magnetic susceptibility anisotropy by using torque balance measurements of excised spinal cord. Rudko et al. (2014) did not find evidence for the presence of a higher-order orientation-dependent term associated to anisotropic susceptibility in fixed rat brain at $9.4\,$T (with TE above $3.84\,$ms) and attributed the observation to confounding contributions of residual background fields and larger ROIs in other studies. Notwithstanding the elaborate evidence for anisotropic myelin susceptibility, Wharton & Bowtell (2013) emphasise that a $\sin^4\theta$-dependence of R_2^* may be produced by WM fibres composed of material of purely isotropic magnetic susceptibility in the short-time regime, and they assign the superposition of $\sin^2\theta$ and $\sin^4\theta$-dependence in their data (post-mortem pig brain at $7\,$T with TE-range of $5\ldots23\,$ms) to an incomplete nulling of the myelin water compartment, which is itself likely to be in the linear regime.

Regarding susceptibility effects from deoxygenated venous blood, it is hypothesised that the preferred orientation of vasculature in WM (being parallel to axons) could be a contributor to the observed orientation-dependence. Bender & Klose (2010) computed that a volume fraction of blood vessels carrying deoxygenated blood of only 1.22% could cause the observed $\sin^2\theta$-dependence. Denk et al. (2011), however, considered this effect to be negligible as venous blood contributes less than 1% to the signal, and they did not observe differences in voxels acquired with different aspect ratios. Gil et al. (2016) suggested that increased vascularisation in regions of larger axonal diameter could further impact

$R^*_{2,\text{aniso}}$.

Iron induces microscopic field inhomogeneities which results in spin dephasing. Lee et al. (2011) stated that iron is not expected to show susceptibility anisotropy due to its spherical shape and sparse distribution, but Oh et al. (2013) suggested that iron may still have contributions to the orientation-dependent R_2^* contrast as iron-positive cells are often found to be aligned along fibres. However, they found that extracting the iron in post-mortem human brain tissue only affected the orientation-dependent R_2^* slightly, indicating that iron is not the primary source for R_2^* anisotropy in the brain white matter.

1.2. T_2 anisotropy and dynamic interactions

The majority of previous studies have reported myelin susceptibility as the primary source of T_2 anisotropy: the random motion of molecules in the induced mesoscopically inhomogeneous magnetic field causes the phase accumulated by the spins prior to the refocusing pulse not to be completely rephased after the refocusing pulse. Oh et al. (2013) found a combined $\cos 2\theta$- and $\cos 4\theta$-dependence (motivated by susceptibility anisotropy) – which is equivalent to a superposition of $\sin^2\theta$ and $\sin^4\theta$ plus a constant term (Appendix B) – in their post-mortem T_2 data at $7\,\text{T}$ with TE-range of $9\ldots 39\,\text{ms}$ and a large range of orientations of the sample w.r.t. $\vec{B}_0$. Gil et al. (2016) demonstrated a preferred $\sin^4\theta$-relationship over a $\sin^2\theta$-relationship for R_2 at $3\,\text{T}$ in the living human brain (TE = $9.6\ldots 96\,\text{ms}$), in agreement with the quadratic regime observed in numerical simulations of cylindrical perturbers with diameters below $15\,\mu\text{m}$ (Marques & Bowtell, 2008). Knight et al. (2017) provided a theoretical derivation from the Bloch-Torrey equation, which predicts a $\sin^4\theta$-dependence for diffusion-mediated decoherence due to susceptibility differences (quadratic regime) and a $\sin^2\theta$-dependence for the interaction between susceptibility differences and applied field gradients.

The mechanism behind the effect of iron on T_2 is less clear; a model of water diffusion through the inhomogeneous magnetic field created by magnetic particles does not fully explain the experimental data (Gossuin et al., 2004; Hernando et al., 2014). Instead, it is thought that relaxation occurs through chemical exchange with protons at the particle surface. The effect of iron on R_2-anisotropy remains to be further elucidated.

1.3. Magic angle effects

The magic-angle effect can be observed in certain tissues with anisotropic microstructure, and in nervous tissue has been attributed to dipole-dipole interactions in ordered collagen fibres (Chappell et al., 2004) or between myelin-bound water protons (Lee et al., 2011). The R_2 has a characteristic $(3\cos^2\theta - 1)^2$ dependence, which can also be written as a superposition of $\sin^2\theta$ and $\sin^4\theta$ with mutually dependent multipliers plus a constant (Appendix B). The results of Oh et al. (2013); Lee et al. (2011) suggested that the magic-angle effect may not be the primary source for R_2-orientation dependence but did not fully exclude its existence. Birkl et al. (2020) find the myelin water fraction derived from CPMG ($3\,\text{T}$, TE > $8\,\text{ms}$) to show an orientation-dependence akin to the magic angle model, but not necessarily

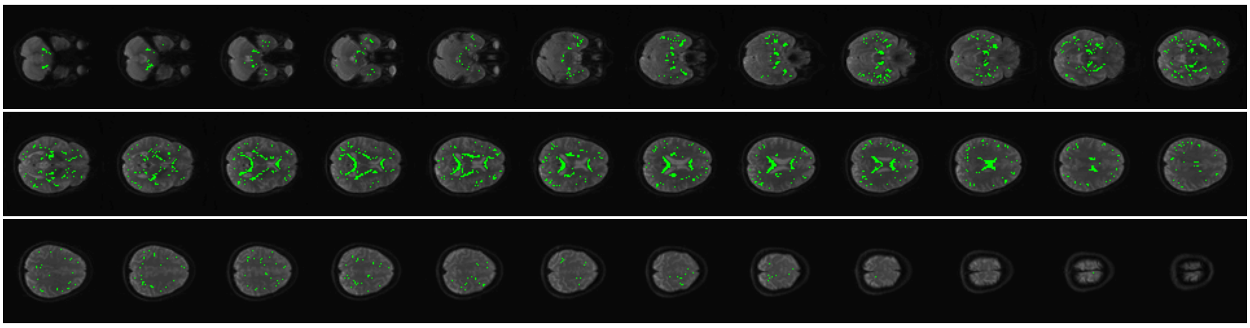

Figure S1: Single fibre populations voxels are shown in green over a b_0, TE = 54 ms brain image.

the myelin water T_2 or the combined intra/extra-cellular water T_2. They speculate on the involvement of microtubule and neurofilaments to cause dipole-dipole coupling effects.

2. Supplementary figures and tables

Figure S1 shows single-fibre-population voxels (in green) mapped over the b_0 brain images.

Figure S2 shows the example data from a single subject acquired with subject's head tilted relative to $\vec{B}_0$.

Table S1 summarises all results from fitting $R_2(\theta)$ with variable isotropic and anisotropic representations.

Figure S3 visualises each of the fit function using the best-fitting parameters from Table S1.

In Figure S4, R_2 values are plotted and analysed as a function of fibre orientation to $\vec{B}_0$ for those SFP voxels with the 'orientation coherence' parameter p_2 above 0.5.

Figure S5 shows an example of T_2-maps corresponding the R_2 maps in Figure 4 of the manuscript.

Figure S6a and Figure S6b show intra- and extra-axonal $R_2(\theta)$-estimates, respectively, along different tracts for all subjects and both head orientations. Table S2 and Table S3 list values shown in barplots in Figure 6 in the main manuscript for mono-exponential and extra-axonal R_2 values, respectively.

In Figure S9 (left column) we estimated the intra- and extra-axonal R_2 values (top and bottom rows, respectively) using the method proposed by McKinnon & Jensen (2019) and analysed them as a function of θ. In the right column of the same figure we included the corresponding plots from this work.

Figure S7 and Figure S8 we provided segment-wise analysis under the reduced influence of fibre-orientational dispersion (by including only those SFP voxels with $p_2 > 0.5$ when calculating segmentwise R_2- and θ-values).

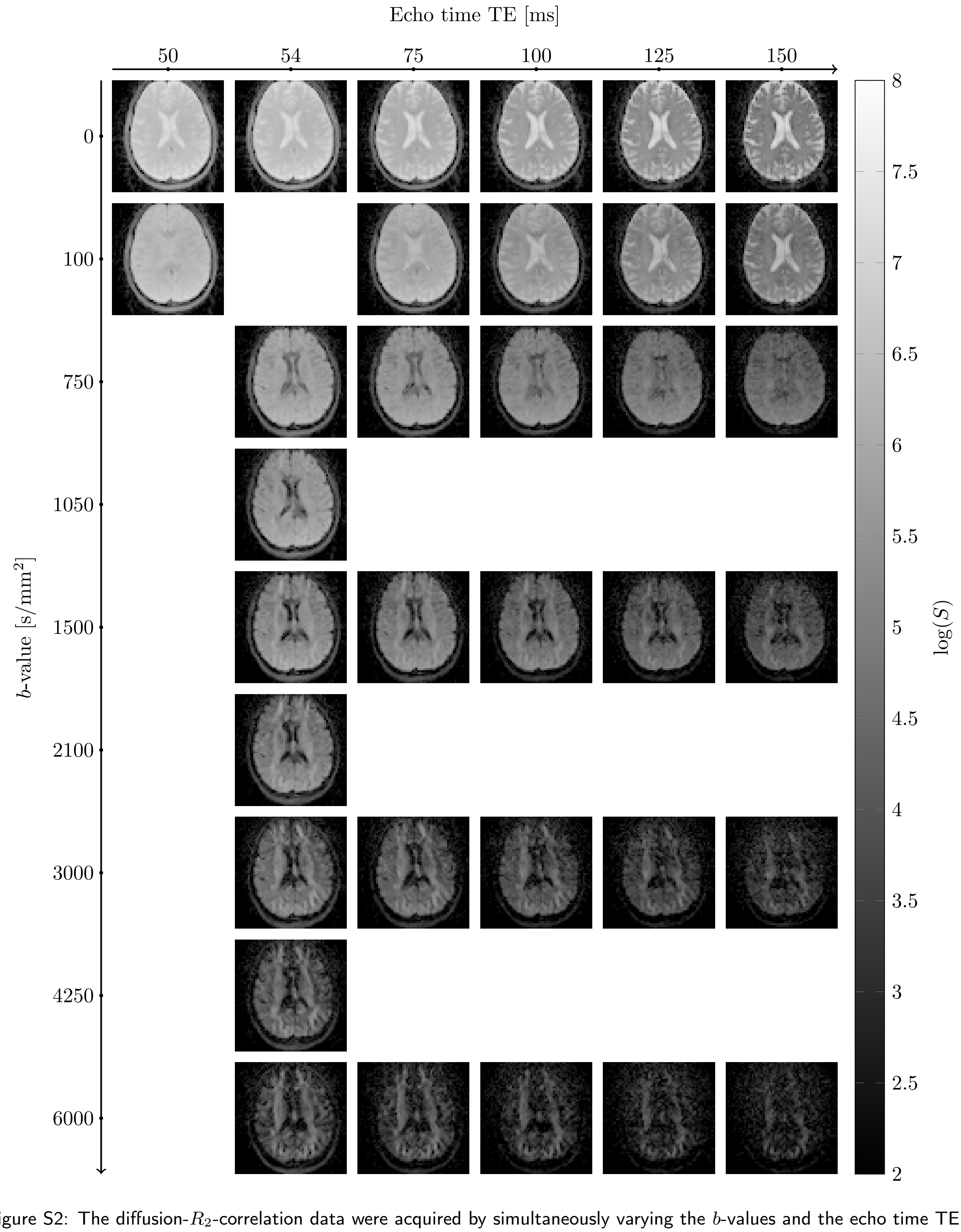

Figure S2: The diffusion-R_2-correlation data were acquired by simultaneously varying the b-values and the echo time TE of the diffusion-weighted spin-echo EPI sequence. The example data were acquired in tilted head orientation with diffusion gradients aligned with the superior-inferior axis.

A. mono-exponential R_2

Fit function	$R_{2,\text{iso}}$ $[\text{s}^{-1}]$	$R_{2,\text{aniso}}$ $[\text{s}^{-1}]$	Δ_{AIC}
$R_{2,\text{iso}}$	15.2	0	2678
$R_{2,\text{iso}} + R_{2,\text{aniso}} \cdot \sin^2\theta$	13.9	2.0	48
$R_{2,\text{iso}} + R_{2,\text{aniso}} \cdot \sin^4\theta$	14.4	1.6	328
$R_{2,\text{iso}} + R_{2,\text{aniso}_1} \cdot \sin^2\theta + R_{2,\text{aniso}_2} \cdot \sin^4\theta$	13.6	3.3 −1.1	0
$R_2(\theta) = R_{2,\text{iso}} + R_{2,\text{aniso}} \cdot \left[1 - \frac{1}{4}(3\cos^2\theta - 1)^2\right]$	13.7	2.0	1332

B. intra-axonal R_2

Fit function	$R_{2,\text{iso}}$ $[\text{s}^{-1}]$	$R_{2,\text{aniso}}$ $[\text{s}^{-1}]$	Δ_{AIC}
$R_{2,\text{iso}}$	12.6	0	76
$R_{2,\text{iso}} + R_{2,\text{aniso}} \cdot \sin^2\theta$	12.4	0.4	42
$R_{2,\text{iso}} + R_{2,\text{aniso}} \cdot \sin^4\theta$	12.5	0.3	58
$R_{2,\text{iso}} + R_{2,\text{aniso}_1} \cdot \sin^2\theta + R_{2,\text{aniso}_2} \cdot \sin^4\theta$	12.0	2.4 −1.7	1
$R_2(\theta) = R_{2,\text{iso}} + R_{2,\text{aniso}} \cdot \left[1 - \frac{1}{4}(3\cos^2\theta - 1)^2\right]$	12.0	0.8	0

C. extra-axonal R_2

Fit function	$R_{2,\text{iso}}$ $[\text{s}^{-1}]$	$R_{2,\text{aniso}}$ $[\text{s}^{-1}]$	Δ_{AIC}
$R_{2,\text{iso}}$	18.7	0	838
$R_{2,\text{iso}} + R_{2,\text{aniso}} \cdot \sin^2\theta$	16.8	2.8	34
$R_{2,\text{iso}} + R_{2,\text{aniso}} \cdot \sin^4\theta$	17.4	2.4	0
$R_{2,\text{iso}} + R_{2,\text{aniso}_1} \cdot \sin^2\theta + R_{2,\text{aniso}_2} \cdot \sin^4\theta$	17.3	0.2 2.3	2
$R_2(\theta) = R_{2,\text{iso}} + R_{2,\text{aniso}} \cdot \left[1 - \frac{1}{4}(3\cos^2\theta - 1)^2\right]$	17.4	1.6	710

Table S1: Transverse relaxation rates R_2 were modelled as a function of white matter fibre orientation θ with respect to the magnetic field vector $\vec{B}_0$.

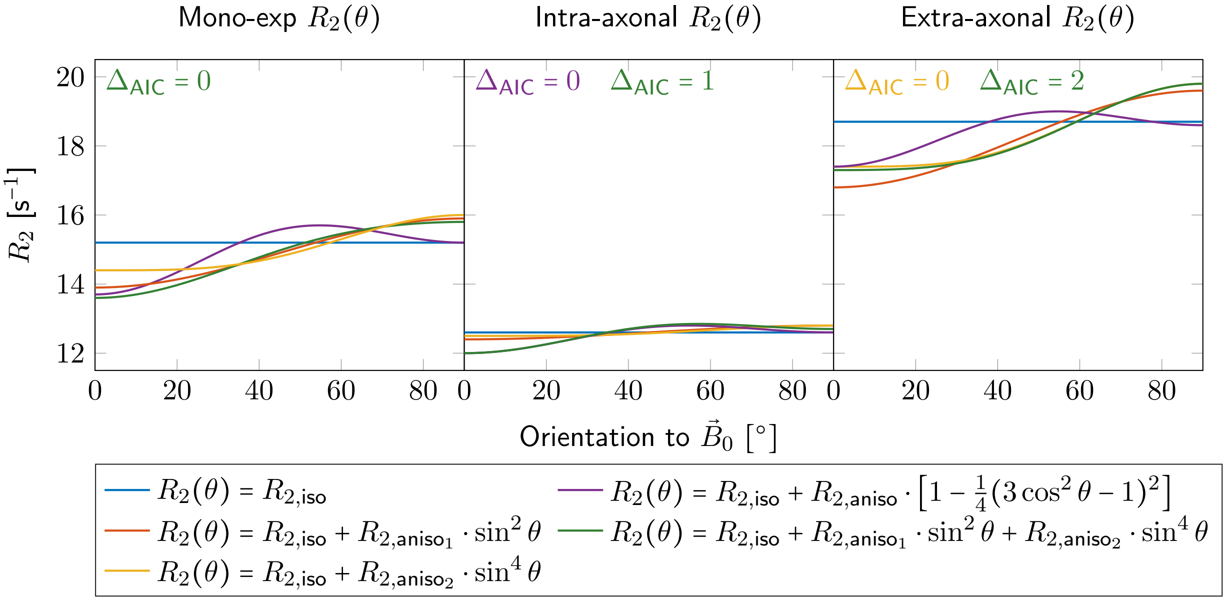

Figure S3: Each of the fit functions is visualised using the best-fitting parameters from Table S1.

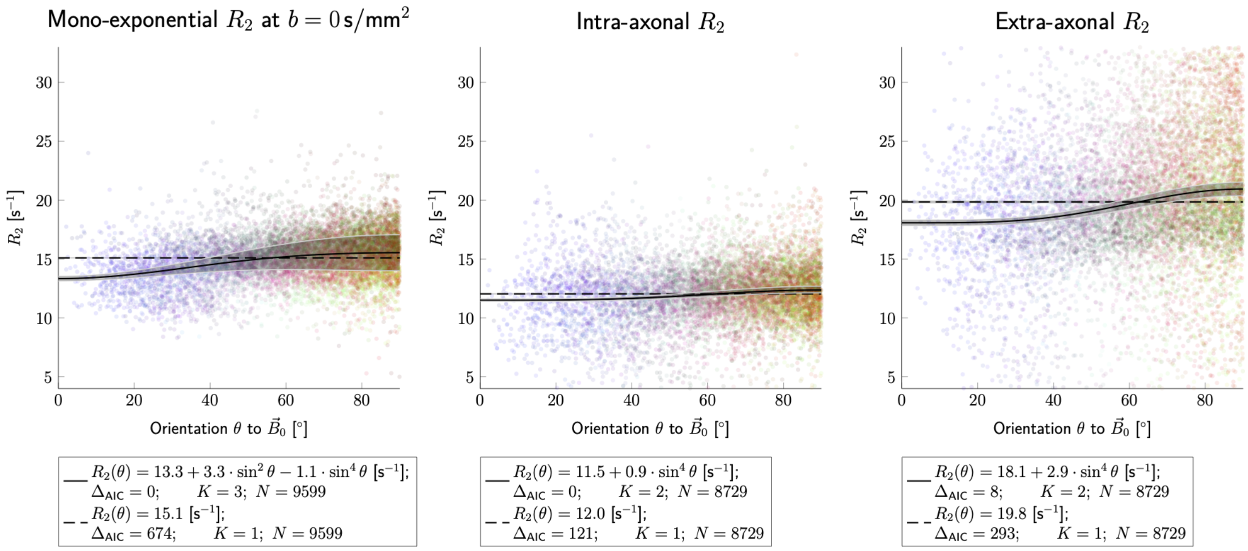

Figure S4: As Figure 4 in the main manuscript, but only voxels with $p_2 > 0.5$ included.

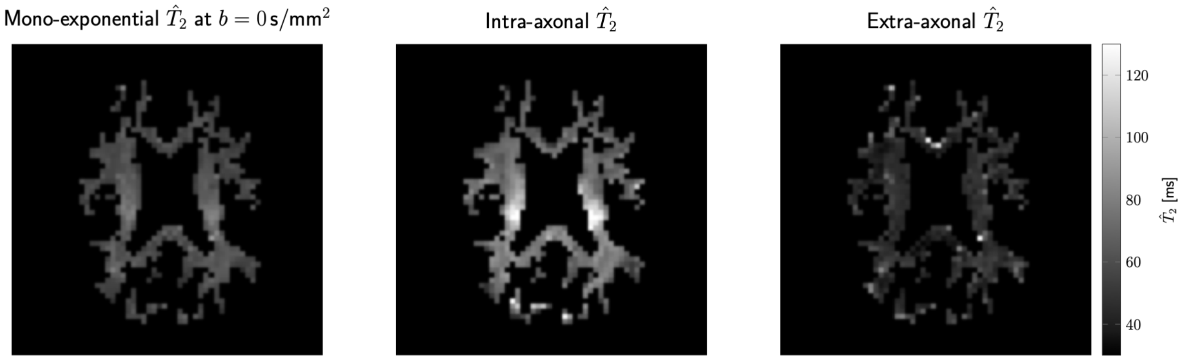

Figure S5: Caption

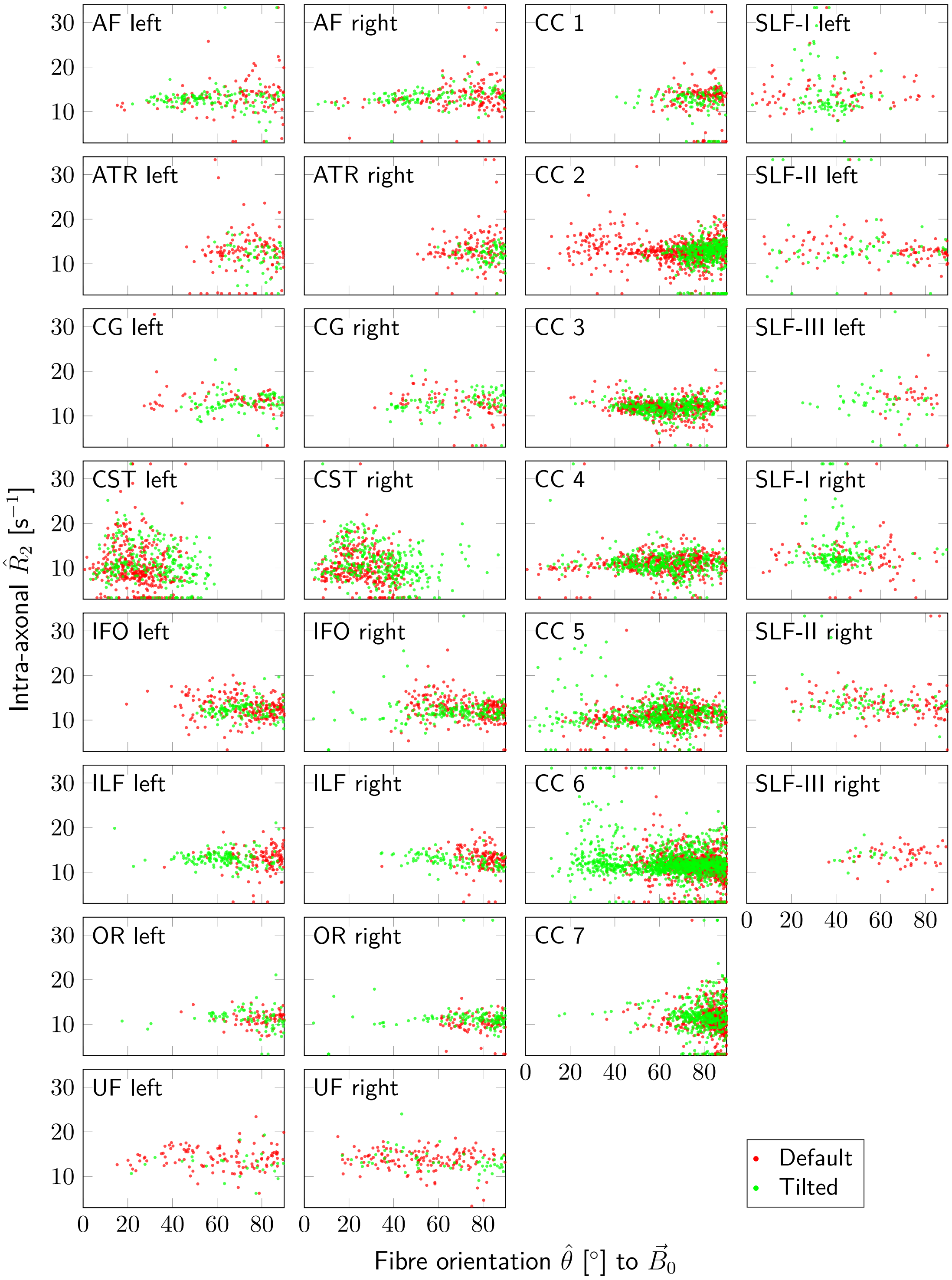

Figure S6a: Intra-axonal relaxation rates $\hat{R}_2$ estimated from diffusion-T_2 correlation data are plotted against fibre orientation $\hat{\theta}$ to the magnetic field $\vec{B}_0$ for default (red) and tilted (green) head orientations. Each point represents one of the SFP voxels from one of 29 fibre tracts (separate plots) in each subject.

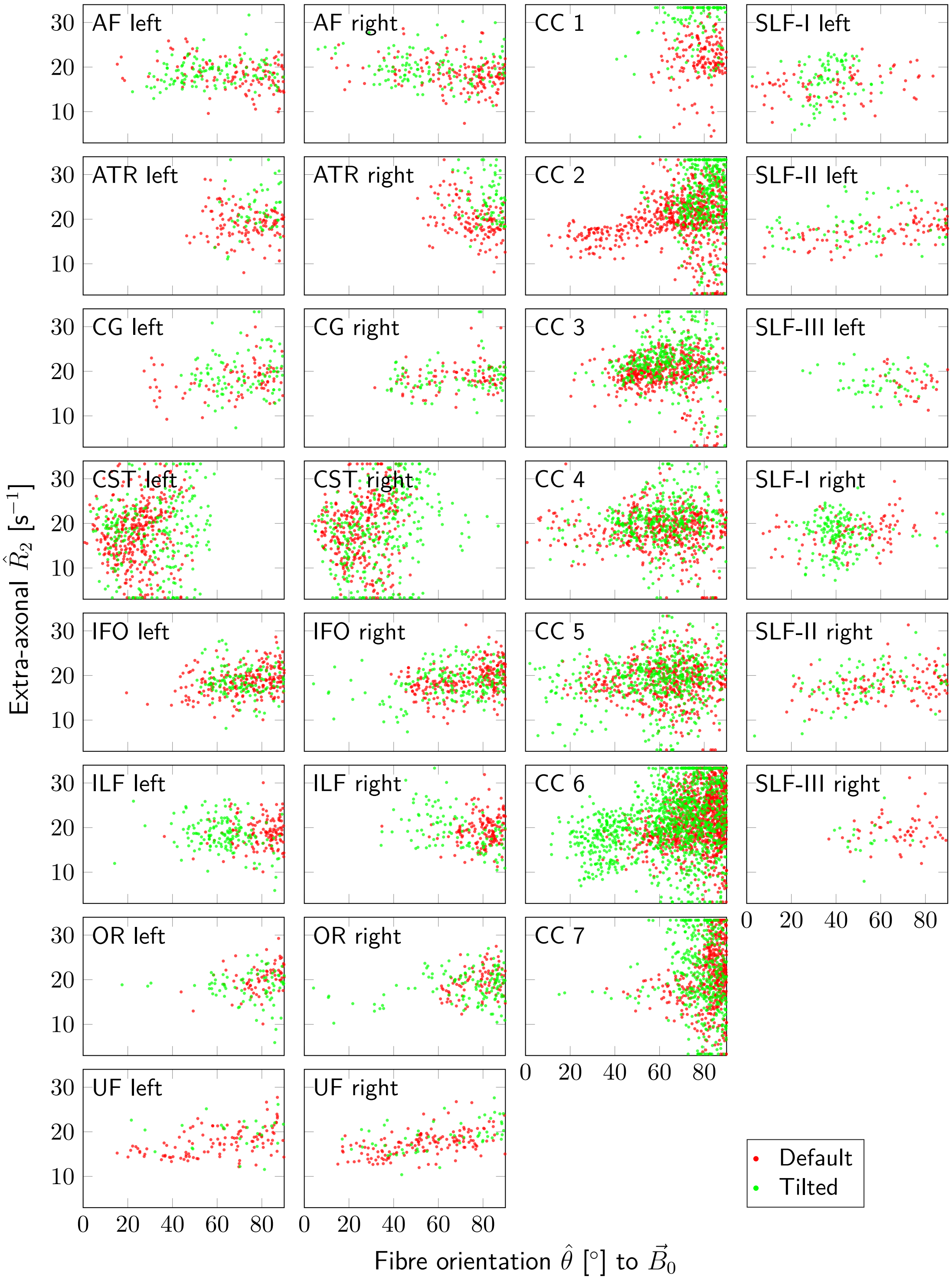

Figure S6b: Extra-axonal relaxation rates $\hat{R}_2$ estimated from diffusion-T_2 correlation data are plotted against fibre orientation $\hat{\theta}$ to the magnetic field $\vec{B}_0$ for default (red) and tilted (green) head orientations. Each point represents one of the SFP voxels from one of 29 fibre tracts (separate plots) in each subject.

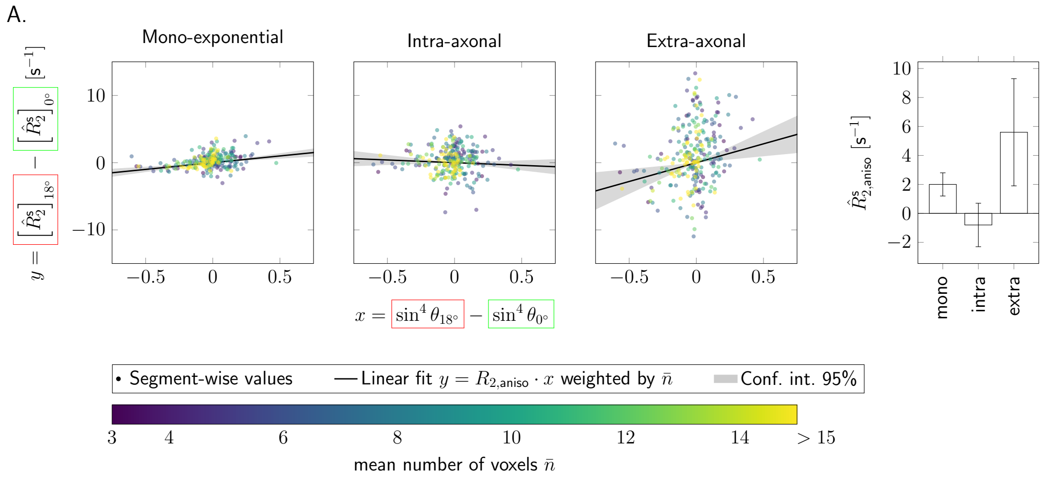

B. Fitting results

	$R_{2,\mathrm{mono}}$ [s^{-1}]	$R_{2,\mathrm{intra}}$ [s^{-1}]	$R_{2,\mathrm{extra}}$ [s^{-1}]
$\sin^2\theta$	2.9 (1.3)	-0.9 (2.3)	7.1 (5.4)
$\sin^4\theta$	2.0 (0.8)	-0.8 (1.5)	5.6 (3.7)

C. Akaike's Information Criterion (AIC), $N = 256$

	ΔAIC($R_{2,\mathrm{mono}}$)	ΔAIC($R_{2,\mathrm{intra}}$)	ΔAIC($R_{2,\mathrm{extra}}$)
$\sin^2\theta$	0	1	0
$\sin^4\theta$	0	1	0
isotropic	17	0	7

Figure S7: Similarly to Figure 7 of the manuscript, SFP voxels were assigned to a segment of a tract if they were wholly contained within that tract segment and their orientation was within 15° of the tangent to the core-streamline in that segment. Additionally, segment-wise analysis was performed under the reduced influence of fibre-orientational dispersion: only those SFP voxels with $p_2 > 0.5$ are included when calculating segment-wise R_2- and θ-values.

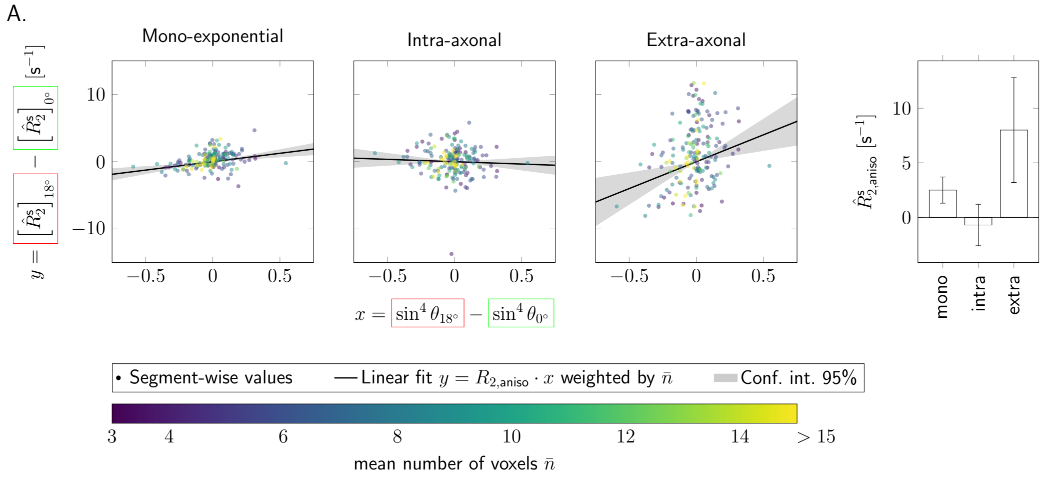

B. Fitting results

	$R_{2,\mathrm{mono}}$ [s^{-1}]	$R_{2,\mathrm{intra}}$ [s^{-1}]	$R_{2,\mathrm{extra}}$ [s^{-1}]
$\sin^2\theta$	3.5 (1.7)	-1.0 (2.7)	10.5 (6.9)
$\sin^4\theta$	2.5 (1.2)	-0.7 (1.9)	8.0 (4.8)

C. Akaike's Information Criterion (AIC), $N = 256$

	ΔAIC($R_{2,\mathrm{mono}}$)	ΔAIC($R_{2,\mathrm{intra}}$)	ΔAIC($R_{2,\mathrm{extra}}$)
$\sin^2\theta$	1	2	2
$\sin^4\theta$	0	1	0
isotropic	14	0	9

Figure S8: SFP voxel assignment to a segment of a tract was restricted further: only those voxels which were oriented within 10° of the tangent to the core-streamline in that segment were used. Additionally, segment-wise analysis was performed under the reduced influence of fibre-orientational dispersion: only those SFP voxels with $p_2 > 0.5$ are included when calculating segment-wise R_2- and θ-values.

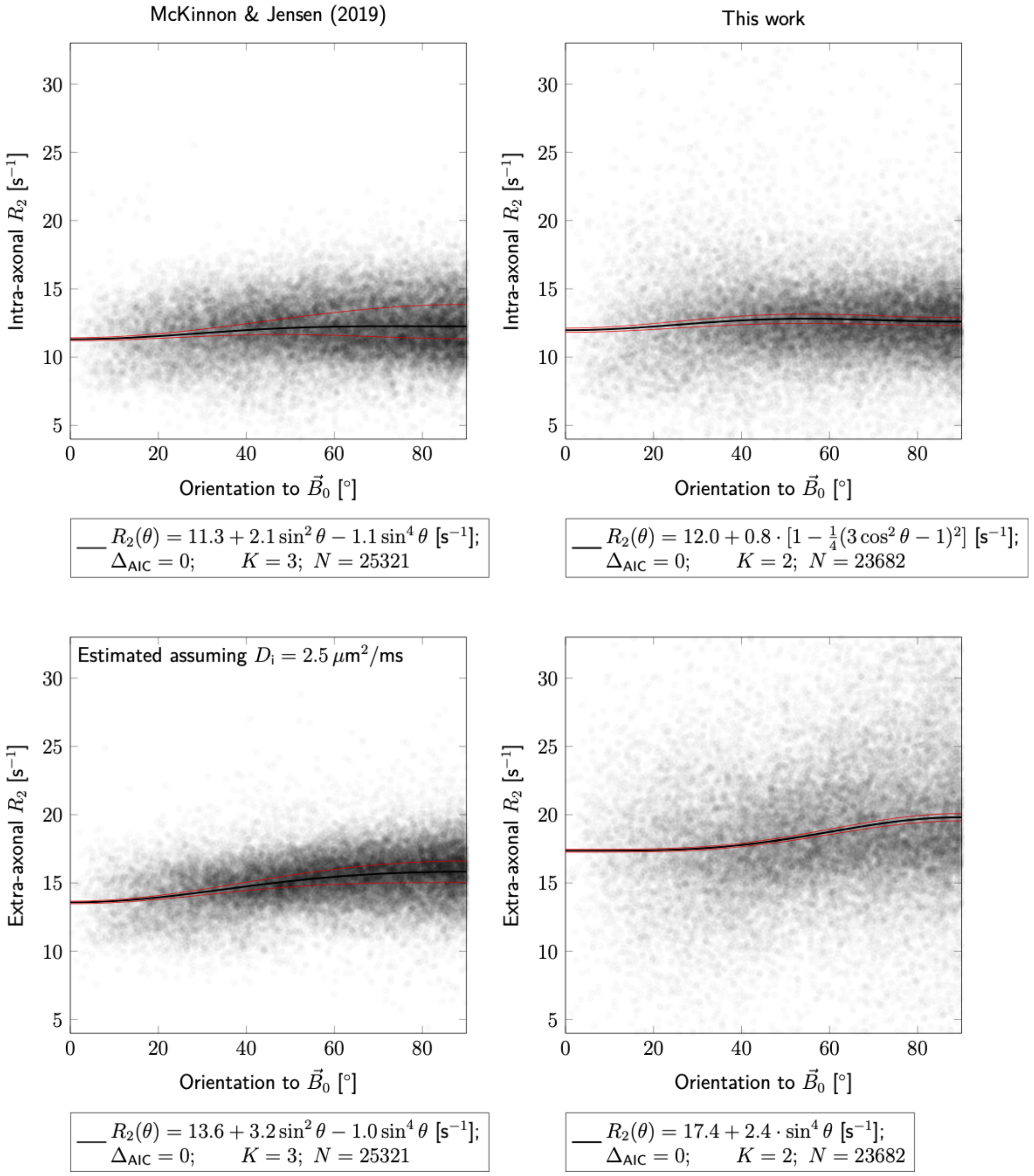

Figure S9: Comparison between the intra-axonal R_2-values estimated using the method introduced by McKinnon & Jensen (2019) (left) with the approach reported in this work (right). Best fitting curves are plotted in black and the corresponding confidence interval boundaries are in red.

We obtained similar intra-axonal $\hat{R}_{2,\text{iso}}$ ($11.3\ [11.2, 11.4]\,\text{s}^{-1}$ vs $12.1\ [11.9, 12.3]\,\text{s}^{-1}$) and $\hat{R}_{2,\text{aniso}}$ ($1.0\ [0.5, 2.4]\,\text{s}^{-1}$ vs $1.1\ [0.6, 1.8]\,\text{s}^{-1}$) with the two approaches on our data. The difference between this work and the reported results of McKinnon & Jensen (2019) may be due to a difference in selection of SFP voxels (from fODFs vs DT-MRI), the fitting (to individual voxel values vs a graphical fit to the mean in their paper), and the changing diffusion time with changing TE in their acquisition, among others. For the sake of completeness we also report the extra-axonal $\hat{R}_2$ estimated per Eq [3] in McKinnon & Jensen (2019) confirming the larger magnitude of $R_{2,\text{aniso}}$ in the extra-axonal space. However, this approach requires fixing D_{i} and its accuracy can affect the accuracy of $\hat{R}_{2,\text{e}}$, which puts this approach "on a less firm foundation" (McKinnon & Jensen, 2019) than the approach for estimating $\hat{R}_{2,\text{i}}$, potentially further explaining the observed difference in $\hat{R}_{2,\text{iso}}$.

Bundle	$R_2(\theta) = R_{2,\text{iso}}$	$R_2(\theta) = R_{2,\text{iso}} + R_{2,\text{aniso}} \sin^2\theta$		$R_2(\theta) = R_{2,\text{iso}} + R_{2,\text{aniso}} \sin^4\theta$	
	$R_{2,\text{iso}}$	$R_{2,\text{iso}}$	$R_{2,\text{aniso}}$	$R_{2,\text{iso}}$	$R_{2,\text{aniso}}$
AF l	15.9 (0.1)	14.0 (0.4)	2.5 (0.5)	14.8 (0.2)	1.8 (0.4)
AF r	16.1 (0.1)	14.7 (0.3)	1.8 (0.4)	15.2 (0.2)	1.4 (0.3)
ATR l	16.6 (0.2)	11.5 (1.4)	5.6 (1.5)	13.8 (0.8)	3.4 (0.9)
ATR r	17.0 (0.2)	10.5 (1.8)	6.9 (1.9)	13.5 (1.0)	4.0 (1.1)
CC 1	17.2 (0.1)	14.5 (1.5)	3.0 (1.6)	15.7 (0.8)	1.7 (0.9)
CC 2	15.8 (0.1)	15.5 (0.4)	0.4 (0.4)	15.6 (0.3)	0.3 (0.3)
CC 3	15.3 (0.1)	14.7 (0.4)	0.8 (0.5)	14.9 (0.2)	0.5 (0.3)
CC 4	14.0 (0.1)	13.6 (0.2)	0.5 (0.3)	13.8 (0.2)	0.4 (0.3)
CC 5	13.9 (0.1)	13.3 (0.2)	0.9 (0.3)	13.5 (0.1)	0.7 (0.2)
CC 6	15.0 (0.0)	14.0 (0.2)	1.1 (0.2)	14.3 (0.1)	0.9 (0.2)
CC 7	14.7 (0.1)	14.2 (0.9)	0.5 (0.9)	14.3 (0.6)	0.5 (0.6)
CG l	15.9 (0.2)	13.5 (0.7)	2.8 (0.8)	14.3 (0.4)	2.2 (0.6)
CG r	16.1 (0.1)	13.8 (0.5)	2.8 (0.6)	14.8 (0.3)	1.9 (0.4)
CST l	13.1 (0.1)	12.8 (0.2)	1.3 (0.7)	12.9 (0.1)	2.6 (1.1)
CST r	13.2 (0.1)	12.6 (0.2)	2.3 (0.6)	12.8 (0.1)	3.5 (0.8)
IFO l	15.6 (0.1)	15.2 (0.7)	0.5 (0.7)	15.4 (0.4)	0.3 (0.5)
IFO r	15.4 (0.1)	14.2 (0.5)	1.5 (0.6)	14.7 (0.3)	1.0 (0.4)
ILF l	15.7 (0.1)	15.2 (0.6)	0.6 (0.7)	15.3 (0.4)	0.5 (0.5)
ILF r	15.8 (0.1)	17.3 (0.9)	-1.6(1.0)	16.8 (0.6)	-1.2 (0.6)
OR l	15.3 (0.3)	13.0 (1.6)	2.6 (1.0)	13.6 (1.1)	2.0 (1.3)
OR r	14.4 (0.2)	13.3 (0.7)	1.2 (0.8)	13.6 (0.5)	1.0 (0.6)
UF l	16.3 (0.2)	14.7 (0.4)	2.1 (0.5)	15.2 (0.3)	1.9 (0.4)
UF r	16.2 (0.1)	14.4 (0.3)	2.8 (0.4)	15.0 (0.2)	2.4 (0.3)
SLF-I l	14.7 (0.2)	14.2 (0.4)	1.5 (0.8)	14.4 (0.3)	1.7 (0.8)
SLF-I r	14.9 (0.1)	14.1 (0.3)	1.7 (0.6)	14.4 (0.2)	1.9 (0.6)
SLF-II l	15.6 (0.1)	14.8 (0.3)	1.3 (0.4)	15.0 (0.2)	1.1 (0.3)
SLF-II r	15.8 (0.2)	14.3 (0.4)	2.1 (0.5)	15.0 (0.3)	1.5 (0.4)
SLF-III l	15.9 (0.2)	15.5 (0.8)	0.4 (1.0)	15.7 (0.5)	0.3 (0.8)
SLF-III r	16.3 (0.3)	14.2 (1.1)	2.6 (1.4)	15.2 (0.7)	1.7 (0.9)

CI includes 0   l&r values are NOT within CI

Table S2: Isotropic and anisotropic components of the mono-exponential R_2 resulting from fitting $R_2(\theta) = R_{2,\text{iso}}$, $R_2(\theta) = R_{2,\text{iso}} + R_{2,\text{aniso}} \sin^2\theta$, and $R_2(\theta) = R_{2,\text{iso}} + R_{2,\text{aniso}} \sin^4\theta$ per tract. The uncertainties of fitted values are shown in round brackets and are based on 85% CI.

Bundle	$R_2(\theta) = R_{2,\text{iso}}$	$R_2(\theta) = R_{2,\text{iso}} + R_{2,\text{aniso}} \sin^2\theta$		$R_2(\theta) = R_{2,\text{iso}} + R_{2,\text{aniso}} \sin^4\theta$	
	$R_{2,\text{iso}}$	$R_{2,\text{iso}}$	$R_{2,\text{aniso}}$	$R_{2,\text{iso}}$	$R_{2,\text{aniso}}$
AF l	18.6 (0.3)	19.4 (0.9)	-1.1 (1.2)	19.3 (0.6)	-1.0 (0.9)
AF r	19.1 (0.3)	21.4 (0.9)	-3.1 (1.1)	20.6 (0.6)	-2.3 (0.9)
ATR l	19.8 (0.4)	17.8 (3.7)	2.2 (4.1)	18.7 (2.1)	1.3 (2.4)
ATR r	21.2 (0.4)	26.7 (5.0)	-6.0 (5.4)	24.3 (2.8)	-3.6 (3.1)
CC 1	23.2 (0.5)	20.3 (5.3)	3.1 (5.7)	21.7 (3.1)	1.7 (3.5)
CC 2	21.3 (0.3)	14.3 (1.1)	8.2 (1.2)	16.6 (0.8)	6.1 (0.9)
CC 3	20.7 (0.2)	19.1 (0.9)	2.1 (1.2)	20.0 (0.6)	1.2 (0.9)
CC 4	19.4 (0.2)	18.4 (0.7)	1.3 (0.9)	18.8 (0.5)	0.9 (0.7)
CC 5	18.9 (0.2)	17.5 (0.7)	1.9 (0.9)	18.1 (0.5)	1.4 (0.7)
CC 6	21.0 (0.2)	14.9 (0.7)	7.3 (0.8)	16.9 (0.5)	5.5 (0.6)
CC 7	20.4 (0.3)	15.5 (3.3)	5.1 (3.5)	17.3 (2.1)	3.4 (2.3)
CG l	18.6 (0.4)	15.5 (1.8)	3.8 (2.2)	16.6 (1.2)	2.8 (1.5)
CG r	18.5 (0.3)	16.4 (1.3)	2.5 (1.5)	17.2 (0.8)	1.8 (1.1)
CST l	17.9 (0.4)	16.7 (0.6)	5.3 (2.2)	17.2 (0.5)	8.7 (3.6)
CST r	18.8 (0.4)	16.2 (0.7)	9.7 (2.0)	17.6 (0.5)	11.1 (2.7)
IFO l	18.7 (0.2)	15.3 (1.5)	3.9 (1.7)	16.8 (0.9)	2.5 (1.1)
IFO r	18.9 (0.2)	14.4 (1.0)	5.4 (1.2)	15.9 (0.7)	4.1 (0.9)
ILF l	18.7 (0.3)	19.8 (1.6)	-1.4 (1.8)	19.4 (1.0)	-1.0 (1.2)
ILF r	19.4 (0.3)	24.2 (2.1)	-5.4 (2.3)	22.4 (1.3)	-3.7 (1.5)
OR l	19.3 (0.4)	17.0 (2.6)	2.6 (2.9)	17.6 (1.8)	2.1 (2.0)
OR r	19.0 (0.3)	14.8 (1.6)	4.7 (1.7)	16.1 (1.2)	3.5 (1.4)
UF l	18.1 (0.4)	14.6 (1.1)	4.9 (1.4)	15.7 (0.8)	4.0 (1.1)
UF r	17.8 (0.3)	14.2 (0.7)	5.6 (0.9)	15.5 (0.5)	4.8 (0.8)
SLF-I l	16.5 (0.4)	15.2 (0.8)	3.3 (1.8)	15.9 (0.6)	2.9 (1.9)
SLF-I r	17.7 (0.3)	16.4 (0.8)	2.7 (1.6)	16.9 (0.5)	2.6 (1.5)
SLF-II l	17.9 (0.4)	15.7 (0.8)	3.6 (1.1)	16.4 (0.6)	3.0 (1.0)
SLF-II r	18.0 (0.4)	14.6 (0.9)	5.0 (1.3)	16.0 (0.7)	3.7 (1.0)
SLF-III l	17.6 (0.4)	19.5 (1.9)	-2.4 (2.4)	18.6 (1.2)	-1.5 (1.7)
SLF-III r	18.8 (0.6)	15.8 (2.8)	3.8 (3.4)	17.2 (1.6)	2.6 (2.3)

CI includes 0   l&r values are NOT within CI

Table S3: Isotropic and anisotropic components of the extra-axonal R_2 resulting from fitting $R_2(\theta) = R_{2,\text{iso}}$, $R_2(\theta) = R_{2,\text{iso}} + R_{2,\text{aniso}} \sin^2\theta$, and $R_2(\theta) = R_{2,\text{iso}} + R_{2,\text{aniso}} \sin^4\theta$ per tract. The uncertainties of fitted values are shown in round brackets and are based on 85% CI.

3. Our data in the context of previous studies

		mono-exp	intra-axonal		extra-axonal	
		$R_{2,\text{aniso}}$ [s^{-1}]	T_2 [ms]	$R_{2,\text{aniso}}$ [s^{-1}]	T_2 [ms]	$R_{2,\text{aniso}}$ [s^{-1}]
Gil et al. (2016)	WM	$0 \ldots 1.5$				
	CST left	$1.0\ [0.5, 1.5]$				
	CST right	$1.3\ [0.5, 2.1]$				
	CG left	$0.9\ [0.7, 1.1]$				
	CG right	$1.3\ [1.0, 1.6]$				
	ILF left	$-0.1\ [-0.2, 0.0]$				
	ILF right	$0.2\ [-0.1, 0.5]$				
Knight et al. (2017)	WM (FA = 0.5)	1.5				
	WM (FA = 0.7)	1.8				
Tax et al. (2017)	WM		80		65	
Veraart et al. (2018)	WM		$70 \ldots 110$		$50 \ldots 65$	
Lampinen et al. (2020)	WM		$69 \ldots 107$		$60 \ldots 68$	
McKinnon & Jensen (2019)	WM		$50 \ldots 110$	$(2.7)^*$	$40 \ldots 70$	
This work	WM ($\theta < 30°$)	$2.1\ [1.7, 2.4]$	$60 \ldots 118$	$0.8\ [0.6, 1.0]$	$29 \ldots 87$	$2.4\ [2.3, 2.6]$
	WM ($\theta > 70°$)		$62 \ldots 102$		$28 \ldots 71$	

Table S4: Values reported in previous studies and in this work are summarised. Method I stands for the the method where were fitting the curve to the voxel-wise values along the tract, Method II involved comparing default vs tilted segment-wise values.

*The magnitude of anisotropy has not been reported by McKinnon & Jensen (2019), but estimated from their Figure 8 by the authors of this manuscript.

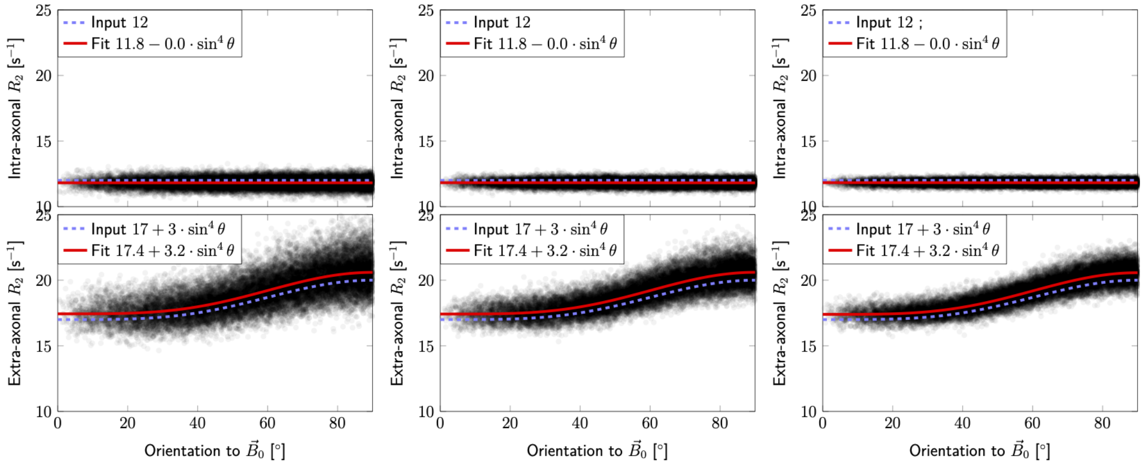

Figure S10: Intra- and exra-axonal $R_2(\theta)$ estimates were explored under the effect of variable SNR levels (from left to right): 70, 100, 130.

4. Simulations

The simulations were performed assuming following default settings (unless stated otherwise):

- We assumed isotropic intra-axonal $R_2(\theta) = 12s^{-1}$ and anisotropic extra-axonal $R_2(\theta) = 17 + 3 \cdot \sin^2\theta$.
- Default diffusion parameters included: signal fraction $f = 0.5$; intra-axonal parallel, and extra-axonal parallel and perpendicular diffusivities $D_{\parallel,\mathrm{i}} = 2.5\mu\mathrm{m}^2/\mathrm{ms}$, $D_{\parallel,\mathrm{e}} = 2\mu\mathrm{m}^2/\mathrm{ms}$, and $D_{\perp,\mathrm{e}} = 0.8\mu\mathrm{m}^2/\mathrm{ms}$, respectively; no orientational dispersion OD $= 0$.
- Gaussian noise was added to the signal with the default SNR input parameters of SNR $= 100$ on the $S(0,0)$ signal corresponding to SNR ≈ 50 on the $S(0,54)$ signal assuming $T_2 \approx 70\,\mathrm{ms}$.

The angles and number of points were taken from the data analysed in this work (cf Figure 4 in the manuscript). These were the default parameters, unless it is stated otherwise.

In Figure S10 we varied the default SNR value to assess the effect of noise on $R_2(\theta)$ estimates.

Figure S11 investigate the minimal detectable difference for the used protocol and SNR of $R_{2,\mathrm{i}}$ and $R_{2,\mathrm{e}}$.

In simulations in Figure S12 we simulated variation of extra-axonal $R_{2,\mathrm{aniso,e}}$ of 0.5, 1, and 2, respectively.

Figure S13 shows simulations where the stick was replaced by a cylinder with a finite radius. In these preliminary results we did not observe a large effect.

References

Bender, B., & Klose, U. (2010). The in vivo influence of white matter fiber orientation towards B_0 on T_2^* in the human brain. *NMR in Biomedicine*, *23*, 1071–1076. doi:10.1002/nbm.1534.

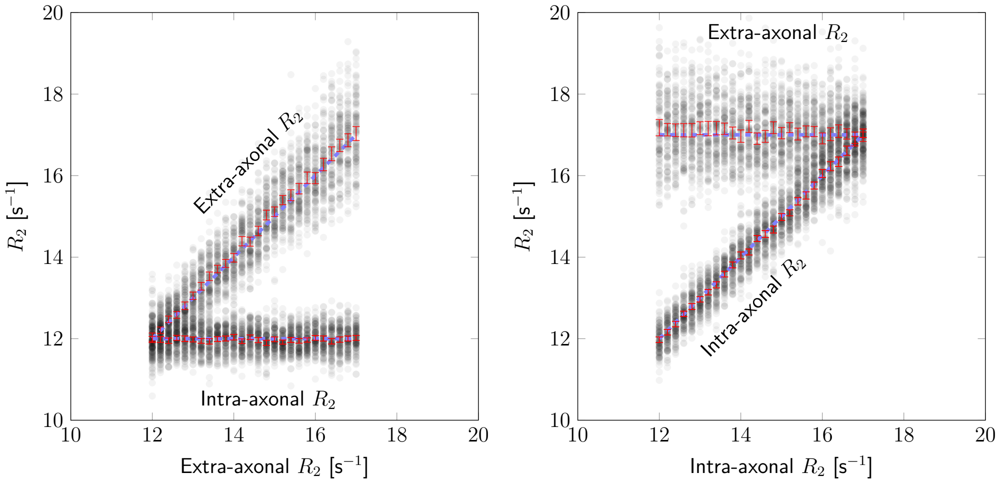

Figure S11: Simulated variation of $R_{2,\mathrm{e}}$ (left), and $R_{2,\mathrm{i}}$ (right) respectively, as described in Appendix A.1. Simulations were performed assuming orientational dispersion of 0.16. 100 noise iterations were realised for each setting of $R_{2,\mathrm{i}}$ and $R_{2,\mathrm{e}}$, and the errorbars give the 95% confidence intervals (CI). CI start overlapping when $R_{2,\mathrm{i}} = 16.8\mathrm{s}^{-1}$ (for $R_{2,\mathrm{e}} = 17\mathrm{s}^{-1}$, right), but not yet when $R_{2,\mathrm{e}} = 12.2\mathrm{s}^{-1}$ (for $R_{2,\mathrm{i}} = 12\mathrm{s}^{-1}$, left).

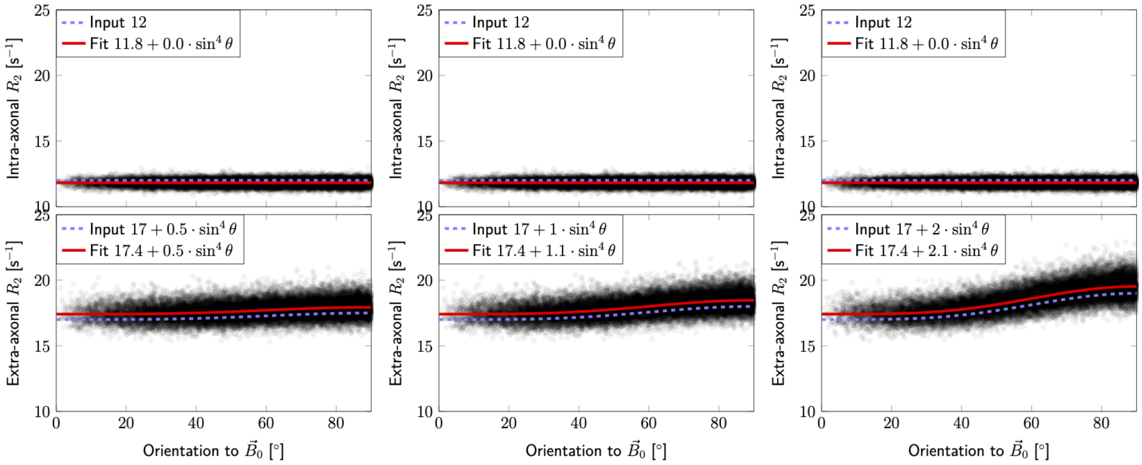

Figure S12: Simulated variation of extra-axonal $R_{2,\mathrm{aniso}}$ of 0.5, 1, and 2 respectively. The confidence intervals for the fits were very narrow (not shown), and did not include 0 for any of the tested $R_{2,\mathrm{aniso}}$.

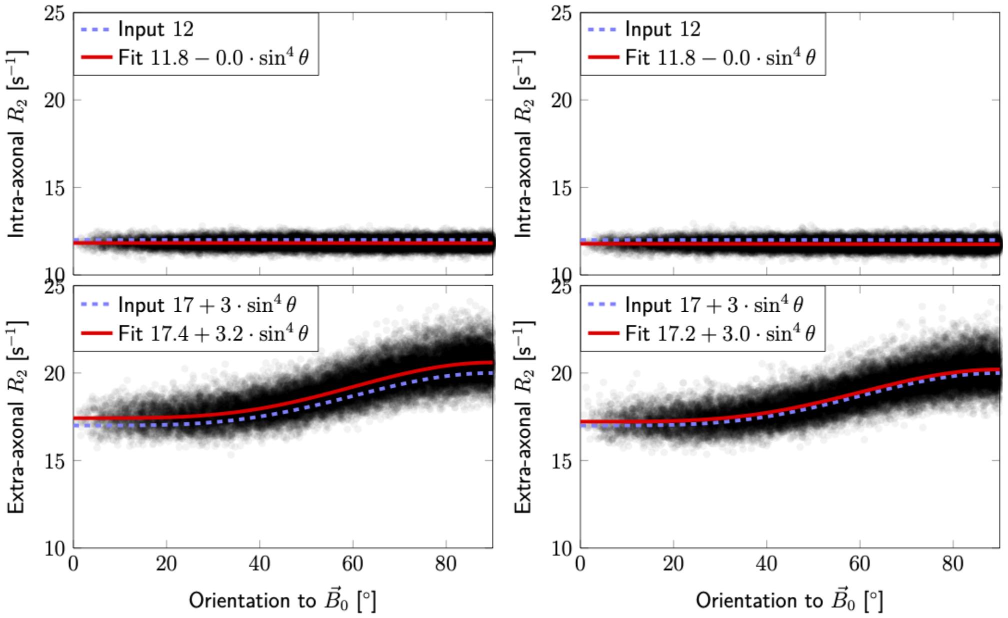

Figure S13: Simulations of a stick (left) and cylinder with radius 2 μm using the MISST toolbox (Drobnjak et al., 2010, plots on the right) The minimal apparent improvement in bias for the extra-axonal compartment in the right column may be due to truncation of SH which cannot describe a delta fibre orientation distribution in the left column, but captures the finite parallel diffusivity in the intra-axonal space as dispersion in the right column. However, the bias in intra-axonal $R_{2,\text{iso}}$ becomes slightly larger.

Birkl, C., Doucette, J., Fan, M., Hernandez-Torres, E., & Rauscher, A. (2020). Myelin water imaging depends on white matter fiber orientation in the human brain. *bioRxiv*, . URL: https://www.biorxiv.org/content/early/2020/03/12/2020.03.11.987925. doi:10.1101/2020.03.11.987925. arXiv:https://www.biorxiv.org/content/early/2020/03/12/2020.03.11.987925.full.pdf.

Chappell, K. E., Robson, M. D., Stonebridge-Foster, A., Glover, A., Allsop, J. M., Williams, A. D., Herlihy, A. H., Moss, J., Gishen, P., & Bydder, G. M. (2004). Magic angle effects in mr neurography. *American Journal of Neuroradiology*, *25*, 431–440. URL: http://www.ajnr.org/content/25/3/431. arXiv:http://www.ajnr.org/content/25/3/431.full.pdf.

Cherubini, A., Péran, P., Hagberg, G. E., Varsi, A. E., Luccichenti, G., Caltagirone, C., Sabatini, U., & Spalletta, G. (2009). Characterization of white matter fiber bundles with <i>T</i> ₂ [*] relaxometry and diffusion tensor imaging. *Magnetic Resonance in Medicine*, *61*, 1066–1072. URL: http://doi.wiley.com/10.1002/mrm.21978. doi:10.1002/mrm.21978.

Denk, C., Torres, E. H., MacKay, A., & Rauscher, A. (2011). The influence of white matter fibre orientation on MR signal phase and decay. *NMR in Biomedicine*, *24*, 246–252. doi:10.1002/nbm.1581.

Dibb, R., & Liu, C. (2017). Joint eigenvector estimation from mutually anisotropic tensors improves susceptibility tensor imaging of the brain, kidney, and heart. *Magnetic Resonance in Medicine*, *77*, 2331–2346. URL: https://onlinelibrary.wiley.com/doi/abs/10.1002/mrm.26321. doi:10.1002/mrm.26321. arXiv:https://onlinelibrary.wiley.com/doi/pdf/10.1002/mrm.26321.

Drobnjak, I., Siow, B., & Alexander, D. C. (2010). Optimizing gradient waveforms for microstructure sensitivity in diffusion-weighted MR. *Journal of Magnetic Resonance*, *206*, 41–51. URL: https://www.sciencedirect.com/science/article/pii/S1090780710001606. doi:10.1016/J.JMR.2010.05.017.

van Gelderen, P., Mandelkow, H., de Zwart, J. A., & Duyn, J. H. (2015). A torque balance mea-

surement of anisotropy of the magnetic susceptibility in white matter. *Magnetic Resonance in Medicine*, *74*, 1388–1396. URL: https://onlinelibrary.wiley.com/doi/abs/10.1002/mrm.25524. doi:10.1002/mrm.25524. arXiv:https://onlinelibrary.wiley.com/doi/pdf/10.1002/mrm.25524.

Gil, R., Khabipova, D., Zwiers, M., Hilbert, T., Kober, T., & Marques, J. P. (2016). An in vivo study of the orientation-dependent and independent components of transverse relaxation rates in white matter. *NMR in Biomedicine*, *29*, 1780–1790. doi:10.1002/nbm.3616.

Gossuin, Y., Muller, R. N., & Gillis, P. (2004). Relaxation induced by ferritin: a better understanding for an improved mri iron quantification. *NMR in Biomedicine*, *17*, 427–432. URL: https://onlinelibrary.wiley.com/doi/abs/10.1002/nbm.903. doi:10.1002/nbm.903. arXiv:https://onlinelibrary.wiley.com/doi/pdf/10.1002/nbm.903.

Hernando, D., Levin, Y. S., Sirlin, C. B., & Reeder, S. B. (2014). Quantification of liver iron with mri: State of the art and remaining challenges. *Journal of Magnetic Resonance Imaging*, *40*, 1003–1021. URL: https://onlinelibrary.wiley.com/doi/abs/10.1002/jmri.24584. doi:10.1002/jmri.24584. arXiv:https://onlinelibrary.wiley.com/doi/pdf/10.1002/jmri.24584.

Knight, M. J., Dillon, S., Jarutyte, L., & Kauppinen, R. A. (2017). Magnetic Resonance Relaxation Anisotropy: Physical Principles and Uses in Microstructure Imaging. *Biophysical Journal*, *112*, 1517–1528. doi:10.1016/j.bpj.2017.02.026.

Lampinen, B., Szczepankiewicz, F., Mårtensson, J., van Westen, D., Hansson, O., Westin, C. F., & Nilsson, M. (2020). Towards unconstrained compartment modeling in white matter using diffusion-relaxation mri with tensor-valued diffusion encoding. *Magnetic Resonance in Medicine*, . URL: http://dx.doi.org/10.1002/mrm.28216. doi:10.1002/mrm.28216.

Lee, J., van Gelderen, P., Kuo, L.-W., Merkle, H., Silva, A. C., & Duyn, J. H. (2011). T_2^*-based fiber orientation mapping. *NeuroImage*, *57*, 225 – 234. doi:10.1016/j.neuroimage.2011.04.026.

Lee, J., Shmueli, K., Fukunaga, M., van Gelderen, P., Merkle, H., Silva, A. C., & Duyn, J. H. (2010). Sensitivity of MRI resonance frequency to the orientation of brain tissue microstructure. *Proceedings of the National Academy of Sciences*, *107*, 5130–5135. doi:10.1073/pnas.0910222107.

Li, W., Wu, B., Avram, A. V., & Liu, C. (2012). Magnetic susceptibility anisotropy of human brain in vivo and its molecular underpinnings. *NeuroImage*, *59*, 2088 – 2097. URL: http://www.sciencedirect.com/science/article/pii/S105381191101202X. doi:https://doi.org/10.1016/j.neuroimage.2011.10.038.

Marques, J. P., & Bowtell, R. W. (2008). Using forward calculations of the magnetic field perturbation due to a realistic vascular model to explore the bold effect. *NMR in Biomedicine*, *21*, 553–565. URL: https://onlinelibrary.wiley.com/doi/abs/10.1002/nbm.1224. doi:10.1002/nbm.1224. arXiv:https://onlinelibrary.wiley.com/doi/pdf/10.1002/nbm.1224.

McKinnon, E. T., & Jensen, J. H. (2019). Measuring intra-axonal T_2 in white matter with direction-averaged diffusion MRI. *Magnetic Resonance in Medicine*, *81*, 2985–2994. URL: http://doi.wiley.com/10.1002/mrm.27617. doi:10.1002/mrm.27617.

Oh, S.-H., Kim, Y.-B., Cho, Z.-H., & Lee, J. (2013). Origin of B_0 orientation dependent $R_2^*(= 1/T_2^*)$ in white matter. *NeuroImage*, *73*, 71 – 79. URL: http://www.sciencedirect.com/science/article/pii/S1053811913000980. doi:doi.org/10.1016/j.neuroimage.2013.01.051.

Rudko, D. A., Klassen, L. M., De Chickera, S. N., Gati, J. S., Dekaban, G. A., & Menon, R. S. (2014). Origins of R_2^* orientation dependence in gray and white matter. *Proceedings of the National Academy of Sciences of the United States of America*, *111*. doi:10.1073/pnas.1306516111.

Tax, C., Rudrapatna, U., Witzel, T., & Jones, D. (2017). Disentangling in two dimensions in the living human brain: Feasbilty of relaxometry-diffusometry using ultra-strong gradients. In *ISMRM* (p. 0838).

Veraart, J., Novikov, D. S., & Fieremans, E. (2018). TE dependent Diffusion Imaging (TEdDI) distinguishes between compartmental T2 relaxation times. *NeuroImage*, *182*, 360–369. URL: https://www.sciencedirect.com/science/article/pii/S1053811917307784?via{%}3Dihub. doi:10.1016/J.NEUROIMAGE.2017.09.030.

Wharton, S., & Bowtell, R. (2012). Fiber orientation-dependent white matter contrast in gradient echo MRI. *Proceedings of the National Academy of Sciences of the United States of America*, *109*, 18559–18564. doi:10.1073/pnas.1211075109.

Wharton, S., & Bowtell, R. (2013). Gradient echo based fiber orientation mapping using R_2^* and frequency difference measurements. *NeuroImage*, *83*, 1011 – 1023. doi:10.1016/j.neuroimage.2013.07.054.

Yablonskiy, D. A., & Haacke, E. M. (1994). Theory of NMR signal behavior in magnetically inhomogeneous tissues: The static dephasing regime. *Magnetic Resonance in Medicine*, *32*, 749–763. URL: https://onlinelibrary.wiley.com/doi/abs/10.1002/mrm.1910320610. doi:10.1002/mrm.1910320610. arXiv:https://onlinelibrary.wiley.com/doi/pdf/10.1002/mrm.1910320610.
